# Noninvasive measurements of skeletal muscle hemodynamics using frequency-domain near-infrared spectroscopy: contributions from adipose and bone tissues

**DOI:** 10.1117/1.BIOS.2.3.035002

**Published:** 2025-09-17

**Authors:** Fatemeh Tavakoli, Angelo Sassaroli, Jodee Frias, Giles Blaney, Sergio Fantini

**Affiliations:** Tufts University, Department of Biomedical Engineering, Medford, Massachusetts, United States

**Keywords:** frequency-domain near-infrared spectroscopy, skeletal muscle, adipose tissue, bone tissue, blood flow, oxygen consumption

## Abstract

**Significance:**

Noninvasive optical assessments of skeletal muscle perfusion and metabolism are crucial for studying muscle physiology. However, measurements can be affected by contributions from overlying adipose and underlying bone tissues.

**Aim:**

We aim to investigate the influence of bone tissue on frequency-domain near-infrared spectroscopy (FD-NIRS) measurements of the human forearm using different data types (intensity and phase), source–detector separations (25 and 37 mm), and acquisition modes (single-distance and dual-slope).

**Approach:**

FD-NIRS data were collected on the forearms of 14 healthy subjects using a custom optical probe designed to regulate probe-skin contact pressure. Tissue blood flow and oxygen consumption were measured with venous and arterial occlusion protocols, respectively. *In vivo* findings were compared with diffusion theory simulations for two- and three-layer media. Ultrasound imaging quantified adipose tissue thickness (1.5 to 9.5 mm) and bone depth (14 to 26 mm).

**Results:**

Experimental results for bone depths below 20 mm could not be reproduced by two-layer simulations, whereas they aligned well with three-layer simulations mimicking adipose, muscle, and bone tissues. Adipose tissue and bone contributions depend on data type, measurement mode, and source–detector separation.

**Conclusions:**

Both adipose and bone tissues may contribute to noninvasive FD-NIRS measurements of skeletal muscle, depending on tissue anatomy and data collection methods.

Statement of Discovery This study demonstrates that, in addition to superficial adipose tissue, deeper bone tissue may also influence noninvasive measurements of skeletal muscle with near-infrared spectroscopy. Careful consideration of contributions from both adipose and bone tissues is essential for accurate optical monitoring of skeletal muscle perfusion and metabolism.

## 1 Introduction

Near-infrared spectroscopy (NIRS) has emerged as a valuable tool in the noninvasive assessment of skeletal muscle hemodynamics, allowing for the study of tissue perfusion and oxygen consumption.^1^ The ability of NIRS to provide data on muscle oxygenation and blood flow cost-effectively and noninvasively offers significant advantages over invasive methods, highlighting its importance in advancing our understanding of muscle physiology.^2^ Furthermore, by incorporating different methods, such as continuous-wave (CW),^3^ time-domain (TD),^4^ and frequency-domain (FD)^5^ techniques, NIRS has provided valuable insights into the complex dynamics of biological tissues under various conditions. For example, in sports settings, NIRS is used to monitor athletes’ muscle oxygenation during training, helping to optimize performance and prevent injuries by identifying muscle fatigue early.^6^ In health monitoring, NIRS can assess muscle function and detect abnormalities in patients with conditions such as chronic obstructive pulmonary disease^7^ and peripheral artery disease,^8^ where understanding muscle oxygenation and blood flow is crucial for managing these diseases.

A major challenge in NIRS measurements of skeletal muscle results from the inhomogeneity of biological tissue, which causes significant contributions to the NIRS signal from superficial layers, such as the skin and adipose tissue.^1^^,^^9^ Numerous studies have demonstrated the necessity of accounting for adipose tissue in NIRS measurements of skeletal muscle and have offered methods for correcting the impact of adipose tissue on NIRS data. Some of these methods rely on calibration measurements that aim to quantify the reduced sensitivity to muscle tissue as adipose tissue thickness increases, based on measurements in a population of subjects with a wide range of adipose tissue thickness,^10^ Monte Carlo simulations,^11^ and/or phantom experiments.^12^ TD measurements have been leveraged to achieve individual adipose tissue thickness corrections based on measurements at four muscle sites^13^ or to perform a quantitative analysis of the mean optical pathlength (which is required for optical measurements of chromophore concentrations in muscle) as a function of tissue optical properties and adipose tissue thickness.^14^ Accordingly, an adipose tissue thickness correction factor was introduced as the ratio between the mean partial optical pathlengths in muscle tissue for a given adipose tissue thickness and for an adipose tissue thickness of 0.^15^

Although a significant amount of attention has been devoted to the role of adipose tissue thickness in NIRS measurements of muscle tissue, to our knowledge, no studies have specifically examined potential contributions from bone tissue, even though its possible impact on NIRS assessments of muscle has been mentioned.^1^ However, we note that NIRS has been used to measure bone hemodynamics in the tibia^16^^–^^18^ or other bones that are close to the tissue surface^19^ because of their accessibility and superficial location. When NIRS is applied to muscle studies, measurements are performed at locations where bone tissue is deep underneath muscle tissue. Therefore, in many cases, bone tissue is assumed not to significantly affect NIRS measurements of muscle tissue. However, considering that the mean optical penetration depth in tissue scales with the square root of the source–detector distance,^20^ there may be cases of relatively thin muscle tissue and/or relatively large source–detector separations for which bone tissue may indeed contribute to the NIRS measurement.

Two key physiological parameters for assessing skeletal muscle with NIRS are blood flow and oxygen consumption, which can be measured using venous and arterial occlusions, respectively, either at rest or during exercise.^9^ Venous occlusion involves inflating a pneumatic cuff to a pressure of typically around 60 mmHg, sufficient to block venous outflow while maintaining arterial inflow. This causes blood to accumulate in the distal tissues at a rate that reflects blood flow. By contrast, arterial occlusion involves inflating the cuff to a pressure (typically around 200 mmHg) that is above systolic pressure, thereby blocking both arterial inflow and venous outflow. This enables the measurement of oxygen consumption through hemoglobin deoxygenation rates. These occlusion protocols provide valuable insights into muscle hemodynamics and oxidative metabolism under varying physiological or pathological conditions.

In this study, we use frequency-domain near-infrared spectroscopy (FD-NIRS) to investigate the potential contribution of bone tissue, employing source–detector separations of 25 and 37 mm for measurements in the human forearm in 14 subjects with bone depth ranging from 14 to 26 mm. FD-NIRS employs a sinusoidally modulated light at frequencies on the order of 100 MHz, allowing for measurement of both the amplitude (intensity) and phase data,^21^ which have distinct spatial sensitivity distributions in tissue (with phase measurements typically probing deeper than intensity).^22^ The absolute optical properties of the tissue, specifically absorption ($\mu_{a}$) and reduced scattering ($\mu_{s}^{\prime }$) coefficients, can be determined by integrating the data from both intensity and phase. Changes in the absorption coefficient ($\mu_{a}$) can be determined by analyzing changes in intensity or phase, which are then translated into changes in oxyhemoglobin (ΔO) and deoxyhemoglobin (ΔD) concentrations associated with muscle hemodynamics.^23^^,^^24^

To collect *in vivo* data, we utilize two source–detector separations of 25 and 37 mm, which further feature different spatial sensitivities at different depths that are associated with adipose, muscle, and bone tissues (with deeper sensitivity for longer separations). In addition, we implement a specialized symmetric source–detector arrangement that allows for both single-distance and dual-slope configurations, with dual-slope offering preferentially deeper sensitivity to the tissue compared with single-distance measurements.^23^^,^^24^ Finally, to interpret the experimental data, we perform theoretical simulations based on diffusion theory for two-layered and three-layered media. This analysis builds upon our previous work, which focused on the baseline optical properties of adipose and muscle tissues and the impact of adipose tissue on muscle tissue measurements.^25^

The primary goal of this study is to determine whether bone tissue, alongside adipose tissue, may impact noninvasive NIRS measurements of skeletal muscle hemodynamics. Although contributions from adipose tissue are unavoidable and must be accounted for,^1^ the impact of bone tissue can potentially be minimized through careful selection of data collection configurations, such as source–detector separations and optical data types (e.g., intensity in CW-NIRS, amplitude or phase in FD-NIRS, or time-of-flight moments in TD-NIRS). Using both *in vivo* measurements and theoretical simulations, this work identifies specific conditions under which bone tissue affects NIRS measurements and provides indications for optimizing FD-NIRS configurations to minimize bone interference in muscle studies.

## 2 Methods

### 2.1 *In Vivo* Measurements on Human Subjects

#### 2.1.1 Experimental setup and FD-NIRS measurements

Fourteen healthy subjects (six male and eight female; age range: 23 to 32 years) participated in this study after providing written informed consent, as approved by the Tufts University Institutional Review Board (IRB). Forearm adipose tissue thickness, muscle tissue thickness, and bone depth were measured using ultrasound imaging [Sonosite S-Nerve, FUJIFILM SonoSite, Inc. (Bothell, Washington, United States)] with a linear transducer array at the same anatomical location as the optical measurements. During ultrasound scanning, subjects were seated comfortably with their forearms resting on a stable desk. Each tissue thickness measurement was performed 3 times, and the mean and standard deviation were calculated for each layer. Table 1 summarizes subject demographics, including age, sex (F, female; M, male), adipose tissue thickness (ATT, corresponding to $L_{1}$ in two- and three-layer simulations), muscle tissue thickness (MTT, corresponding to $L_{2}$ in three-layer simulations), and bone depth (calculated as adipose tissue thickness + muscle tissue thickness, i.e., $L_{1}+L_{2}$ in three-layer simulations).

**Table 1:** Summary of subject demographics and tissue thicknesses measured by ultrasound. Values represent mean ± standard deviation from three independent measurements. F, female; M, male; ATT, adipose tissue thickness; and MTT, muscle tissue thickness.

No.	Sex	Age (yr)	ATT (mm)	MTT (mm)	Bone depth (mm)
1	F	28	3.0 ± 0.2	11.0 ± 0.2	14.0 ± 0.4
2	F	30	4.0 ± 0.1	10.5 ± 0.2	14.5 ± 0.3
3	M	24	2.5 ± 0.2	12.5 ± 0.3	15.0 ± 0.5
4	F	27	3.7 ± 0.1	13.3 ± 0.2	17.0 ± 0.3
5	F	28	4.5 ± 0.2	14.0 ± 0.1	18.5 ± 0.3
6	F	26	6.5 ± 0.3	13.0 ± 0.3	19.5 ± 0.6
7	F	25	7.0 ± 0.5	13.5 ± 0.1	20.5 ± 0.6
8	F	23	6.2 ± 0.2	14.5 ± 0.3	20.7 ± 0.5
9	F	32	5.0 ± 0.2	16.0 ± 0.2	21.0 ± 0.4
10	M	26	1.7 ± 0.2	19.8 ± 0.3	21.5 ± 0.5
11	M	25	1.5 ± 0.1	20.5 ± 0.5	22.0 ± 0.6
12	M	24	2.0 ± 0.2	21.5 ± 0.3	23.5 ± 0.5
13	M	23	9.5 ± 0.5	15.0 ± 0.2	24.5 ± 0.7
14	M	28	8.0 ± 0.4	18.0 ± 0.1	26.0 ± 0.5

**Fig. 1:**
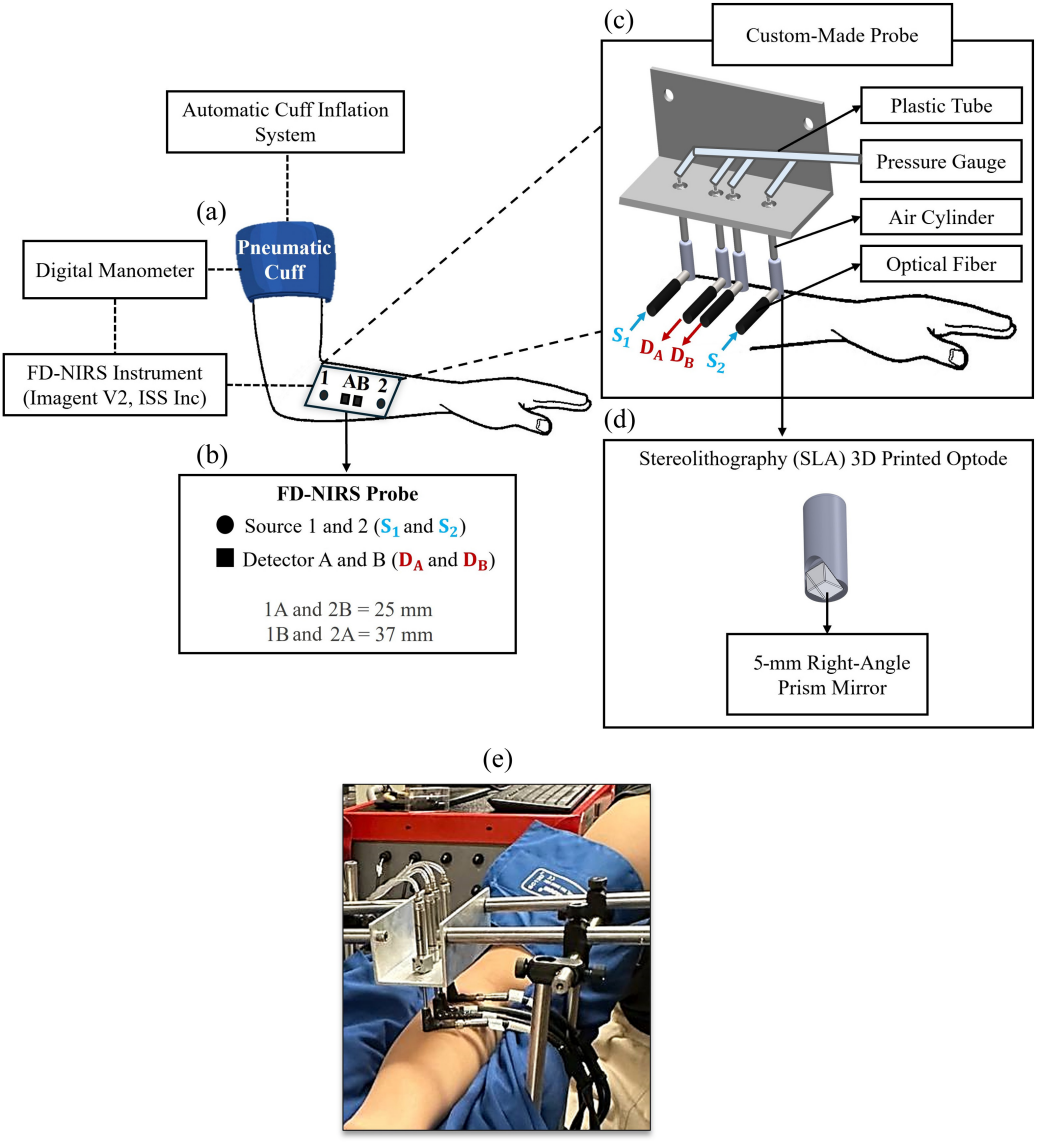
Experimental setup for FD-NIRS measurements on the human forearm. (a) Pneumatic arm cuff [SC12D, D.E. Hokanson, Inc. (Bellevue, Washington, United States)] placed on the dominant arm and connected to a digital manometer [Series 626 Pressure Transmitter, Dwyer Instruments Inc. (Michigan City, Indiana, United States)]. The cuff is regulated by an automated inflation system [E-20 Rapid Cuff Inflation System, D.E. Hokanson, Inc. (Bellevue, Washington, United States)] to maintain stable pressures of 60 mmHg for venous occlusion and 200 mmHg for arterial occlusion. The mechanical design responds rapidly to contact pressure fluctuations, maintaining constant force via a compliant structure rather than active feedback. (b) Optical probe connected to the FD-NIRS system [Imagent V2, ISS Inc. (Champaign, Illinois, United States)], positioned over the brachioradialis muscle. The probe includes two dual-wavelength illumination fibers (sources 1 and 2) and two sets of collection fiber bundles (detectors A and B), enabling two short single-distance measurements at 25 mm (1A and 2B), two long single-distance measurements at 37 mm (1B and 2A), and one dual-slope measurement (1AB2). (c) Custom-made probe housing with two source optodes and two detector optodes integrated with a pressure regulator to control contact pressure on the tissue, eliminating the need for bandage wraps. (d) SLA 3D-printed optode with cylindrical housing, a 5-mm right-angle prism mirror [Thorlabs (Newton, New Jersey, United States)] to redirect light by 90 deg, and openings for optical fiber and round-body air cylinder [Double-Acting, Universal Mount (Robbinsville, New Jersey, United States)] with an 8-mm bore and 76-mm stroke. (e) Photograph of the FD-NIRS setup positioned on the subject’s forearm.

To perform venous and arterial occlusion protocols, participants were seated comfortably with their dominant forearm resting on a flat surface. A pneumatic arm cuff [SC12D, D.E. Hokanson, Inc. (Bellevue, Washington, United States)] was placed around the upper arm, as illustrated in Fig. 1(a). The cuff was inflated using an automated system [E-20 Rapid Cuff Inflation System, D.E. Hokanson, Inc. (Bellevue, Washington, United States)] with an inflation time of less than 0.5 s. During venous occlusion, the cuff pressure was set to 60 mmHg, whereas arterial occlusion was performed at 200 mmHg. Cuff pressure was continuously monitored using a digital manometer [Series 626 Pressure Transmitter, Dwyer Instruments Inc. (Michigan City, Indiana, United States)] and recorded simultaneously with FD-NIRS data. The venous occlusion protocol consisted of a 1-min baseline, 1-min occlusion, and a 1-min recovery period. The arterial occlusion protocol included a 2-min baseline, 2-min occlusion, and a 2-min recovery period.

Optical measurements were performed using an FD-NIRS instrument [Imagent, ISS Inc. (Champaign, Illinois, United States)] operating with sinusoidally modulated light at 140 MHz and wavelengths of 690 and 830 nm. An optical probe connected to the instrument was positioned over the brachioradialis muscle on the subject’s forearm. This probe included two dual-wavelength illumination fibers (sources 1 and 2) and two collection fiber bundles (detectors A and B). Data were acquired at a sampling rate of 2.4 Hz.

Figure 1(b) shows the optical probe, which enabled multiple measurement configurations, including two short single-distance measurements at 25 mm (1A and 2B), two long single-distance measurements at 37 mm (1B and 2A), and a dual-slope measurement (1AB2). The dual-slope configuration used a symmetric arrangement of two sources and two detectors to measure the average of two paired slopes of FD-NIRS data (intensity or phase) versus source–detector separation.^23^^,^^24^ Baseline optical properties of the subject’s forearm, specifically $\mu_{a}$ and $\mu_{s}^{\prime }$ for each subject and vascular occlusion protocol, were obtained from dual-slope intensity and phase data by assuming tissue homogeneity and applying a self-calibrated method^26^ and are reported in Table S1 of the Supplementary Material. FD-NIRS data, namely intensity and phase measured at each wavelength in single-distance and dual-slope configurations, were converted into absorption changes ($\Delta \mu_{a}$) using a modified Beer–Lambert law adapted to each data type,^27^ by accounting for the baseline optical properties.

Finally, $\Delta \mu_{a}$ at 690 and 830 nm, derived from each data type, were converted into ΔO and ΔD using Beer’s law and the molar extinction coefficients of oxy- and deoxyhemoglobin at the two wavelengths employed in this study. It is important to note that, under the protocols used here, the observed hemodynamic changes are primarily attributed to hemoglobin as myoglobin concentration and oxygen saturation remain largely unaffected by vascular occlusion interventions performed at rest.^1^

#### 2.1.2 Implementation of the optical probe for consistent measurements on the forearm tissue

A critical requirement for NIRS measurements of muscle tissue is maintaining adequate and consistent contact pressure between the optical probe and the skin. Insufficient contact pressure can increase susceptibility to motion artifacts, whereas excessive pressure or securing the probe with tightly wrapped bandages may alter local hemodynamics and confound measurements of blood accumulation and oxygenation. In addition, unstable contact pressure due to improper probe attachment can introduce signal variability originating from the instrumentation rather than the tissue itself. To address these challenges, we developed a custom-built system that eliminates the need for bandage wraps while providing precise control over optode pressure on the skin [see Fig. 1(c)]. The system incorporates specialized optode housing with pneumatically regulated cylinders and an optical-fiber-coupled prism. This design ensures consistent and controllable pressure across all measurements and subjects, thereby improving the reliability and reproducibility of the collected data.

The design process began with the development of detailed technical drawings in SolidWorks computer-aided design software. Each component, including the optode housing, optical prism, and air cylinder mount, was carefully modeled to meet both functional and structural requirements. The optode housing features a cylindrical geometry with two key openings: one for connecting the source or detector optical fibers and another for mounting a round-body air cylinder [Double-Acting, Universal Mount (Robbinsville, New Jersey, United States)] with an 8-mm bore and a 76-mm stroke. A 5-mm right-angle prism mirror [Thorlabs (Newton, New Jersey, United States)] was positioned at the base of the optode to redirect light by 90 deg, enabling efficient coupling of light either from the source fibers into the tissue or from the tissue into the detector fibers [see Fig. 1(d)]. The prism, optical fibers, and air cylinder were fully integrated within the optode housing to prevent misalignment and maintain stable contact with the skin. Once finalized in SolidWorks, the optode housing was fabricated using stereolithography (SLA) 3D printing.

Pressure was applied through a mechanism in which the cylinder stroke interfaced with the optode via a dedicated opening at the top of the housing. Each cylinder was connected to a pressure regulator using flexible plastic tubing, which, in turn, was linked to an automated inflation system [E-20 Rapid Cuff Inflation System, D.E. Hokanson, Inc. (Bellevue, Washington, United States)]. The entire assembly was positioned on the subject’s forearm, and pressure was regulated via the automated inflation system. The applied force was continuously monitored through the pressure regulator to maintain consistent force levels. A minimum pressure of 78 kPa, equivalent to the pressure exerted by an 8 g mass over a horizontal surface with an area of $1\;{\mathrm{mm}}^{2}$, was established to secure the optode against the skin without excessive compression, thereby preventing optode movement during measurements. This design ensured an even distribution of pressure across the optode’s contact surface, minimizing the risk of tissue compression or deformation. The same pressure level was maintained across all subjects and experiments, accommodating differences in forearm size and tissue properties. This adjustable system enhanced the uniformity of FD-NIRS measurements and improved the reliability of the collected data. Figure 1(e) shows a photograph of the FD-NIRS setup on the forearm.

#### 2.1.3 Measurements of blood flow and oxygen consumption

Tissue blood flow measurements were performed using a venous occlusion protocol. Blood flow (BF) was calculated from the initial rate of increase in total hemoglobin concentration (ΔT) following the onset of venous occlusion, as follows:

$$\text{Blood Flow}\;(\mathrm{BF})={\frac{1}{\mathrm{ctHb}}\frac{d}{dt}(\Delta T)|}_{0},$$ (1)

where ctHb is the hemoglobin concentration in blood, assumed to be 15  g/dL (equivalent to 2.3 mM) in this study.^28^ The subscript 0 indicates that the derivative was computed over a time window immediately after the onset of venous occlusion. The initial rate of increase in ΔT was determined by applying a linear fit to ΔT as a function of time following the venous occlusion onset over the initial linear portion of the response (10 s in this study). As noted previously, under resting conditions during venous occlusion, the increase in heme concentration is attributed solely to hemoglobin, allowing myoglobin contributions to d(ΔT)/dt to be neglected. It is important to emphasize that this approach provides an indirect measure of blood flow by assessing the local rate of increase in blood volume. Several factors, such as blood redistribution within the distal portion of the occluded limb, local vascular anatomy, vascular compliance, and the mechanical properties of the probed tissue, can influence the temporal rate of change in local blood volume and introduce potential confounding effects. Therefore, in this study, blood flow should be interpreted as a measure of the local rate of increase in hemoglobin concentration in response to venous occlusion.

Tissue oxygen consumption measurements were performed using an arterial occlusion protocol. Oxygen consumption (OC) was estimated from the initial rates of change in deoxyhemoglobin and oxyhemoglobin concentrations (ΔD and ΔO, respectively) measured immediately after the onset of arterial occlusion, as follows:

$$\text{Oxygen Consumption}\;(\mathrm{OC})={4\frac{d}{dt}(\frac{\Delta D-\Delta O}{2})|}_{0},$$ (2)

where the factor 4 accounts for the four oxygen-binding sites in hemoglobin, and the subscript 0 indicates that the derivative was evaluated over a time window immediately following the initiation of arterial occlusion. The initial rates of ΔD increase and ΔO decrease were determined by applying a linear fit to ΔD and ΔO as a function of time after the occlusion onset over the initial linear portion of the response (60 s in this study).

### 2.2 Theoretical Simulations for Two-Layer and Three-Layer Media

The experimental FD-NIRS data provided measurements of ΔD, ΔO, and ΔT under the assumption that the investigated tissue behaves as a homogeneous, semi-infinite medium. However, this is a strong simplification that does not account for actual tissue heterogeneity. As a result, the measurements yield effective values for ΔD, ΔO, and ΔT. To aid interpretation of the experimental data, we performed simulations based on diffusion theory for two-layer media (representing adipose and muscle tissues) and three-layer media (representing adipose, muscle, and bone tissues).^29^ These simulations provide insight into how tissue heterogeneity affects blood flow and oxygen consumption measurements obtained with FD-NIRS.

The first step in defining the two- or three-layer models involved assigning baseline optical properties, specifically $\mu_{a}$ and $\mu_{s}^{\prime }$ at both wavelengths, as well as the thickness of each layer. The bottom layer in both models was assumed to extend infinitely. This step established the anatomical and optical conditions of the tissue regions being modeled. Next, $\Delta \mu_{a}$ values for each layer at both wavelengths were assigned to simulate changes in hemoglobin concentration and oxygen saturation within the tissue layers. This step captured physiological responses to venous and arterial occlusion, such as hemoglobin accumulation during venous occlusion and hemoglobin desaturation during arterial occlusion for the various tissue layers. In this study, we focused on analyzing the ratios of blood flow and oxygen consumption derived from different FD-NIRS data types (assuming tissue homogeneity) across scenarios involving varied baseline optical properties, simulated hemodynamic responses, and hemoglobin deoxygenation within the tissue layers.

The FD-NIRS data types considered in this study include single-distance intensity (SDI), single-distance phase (SDϕ), dual-slope intensity (DSI), and dual-slope phase (DSϕ). To simulate blood flow and oxygen consumption ratios for these data types (e.g., comparing data types x and y, which may represent either single-distance or dual-slope measurements), we calculated the ratio of effective ΔT between two data types ($\Delta T_{y}/\Delta T_{x}$) and the ratio of effective (ΔD−ΔO) between two data types $(\Delta D-\Delta O{)}_{y}/(\Delta D-\Delta O{)}_{x}$, respectively. In the case of two-layer media, these ratios are expressed as follows:

$$\frac{\Delta T_{y}}{\Delta T_{x}}=\frac{a^{-}[S_{1,y}(\lambda_{1})\Delta \mu_{a,1}(\lambda_{1})+S_{2,y}(\;\lambda_{1})\Delta \mu_{a,2}(\lambda_{1})]+b^{-}[S_{1,y}(\lambda_{2})\Delta \mu_{a,1}(\lambda_{2})+S_{2,y}(\lambda_{2})\Delta \mu_{a,2}(\lambda_{2})]}{a^{-}[S_{1,x}(\lambda_{1})\Delta \mu_{a,1}(\lambda_{1})+S_{2,x}(\lambda_{1})\Delta \mu_{a,2}(\lambda_{1})]+b^{-}[S_{1,x}(\lambda_{2})\Delta \mu_{a,1}(\lambda_{2})+S_{2,x}(\lambda_{2})\Delta \mu_{a,2}(\lambda_{2})]},$$ (3)

$$\frac{(\Delta D-\Delta O{)}_{y}}{(\Delta D-\Delta O{)}_{x}}=\frac{a^{+}[S_{1,y}(\lambda_{1})\Delta \mu_{a,1}(\lambda_{1})+S_{2,y}(\lambda_{1})\Delta \mu_{a,2}(\lambda_{1})]+b^{+}[S_{1,y}(\lambda_{2})\Delta \mu_{a,1}(\lambda_{2})+S_{2,y}(\lambda_{2})\Delta \mu_{a,2}(\lambda_{2})]}{a^{+}[S_{1,x}(\lambda_{1})\Delta \mu_{a,1}(\lambda_{1})+S_{2,x}(\lambda_{1})\Delta \mu_{a,2}(\lambda_{1})]+b^{+}[S_{1,x}(\lambda_{2})\Delta \mu_{a,1}(\lambda_{2})+S_{2,x}(\lambda_{2})\Delta \mu_{a,2}(\lambda_{2})]},$$ (4)

where $S_{k,x}(\lambda_{i})$ and $S_{k,y}(\lambda_{i})$ represent the sensitivities of data types x and y, respectively, to the k’th layer (k=1,2) at the i’th wavelength ($\lambda_{i}$, i=1,2), and $\Delta \mu_{a,k}(\lambda_{i})$ is the change in absorption coefficient in the k’th layer at the i’th wavelength $\lambda_{i}$. The coefficients $a^{\pm }$ and $b^{\pm }$, which depend on $\lambda_{i}(i=1,2)$, are defined as

$$a^{\pm }(\lambda_{1},\lambda_{2})=\frac{\varepsilon_{D}(\lambda_{2})\pm \varepsilon_{O}(\lambda_{2})}{\varepsilon_{O}(\lambda_{1})\varepsilon_{D}(\lambda_{2})-\varepsilon_{O}(\lambda_{2})\varepsilon_{D}(\lambda_{1})},$$ (5)

$$b^{\pm }(\lambda_{1},\lambda_{2})=\frac{\varepsilon_{O}(\lambda_{1})\pm \varepsilon_{D}(\lambda_{1})}{\varepsilon_{O}(\lambda_{1})\varepsilon_{D}(\lambda_{2})-\varepsilon_{O}(\lambda_{2})\varepsilon_{D}(\lambda_{1})},$$ (6)

where $\varepsilon_{O}$ and $\varepsilon_{D}$ denote the molar extinction coefficients of oxyhemoglobin (O) and deoxyhemoglobin (D), respectively.

**Fig. 2:**
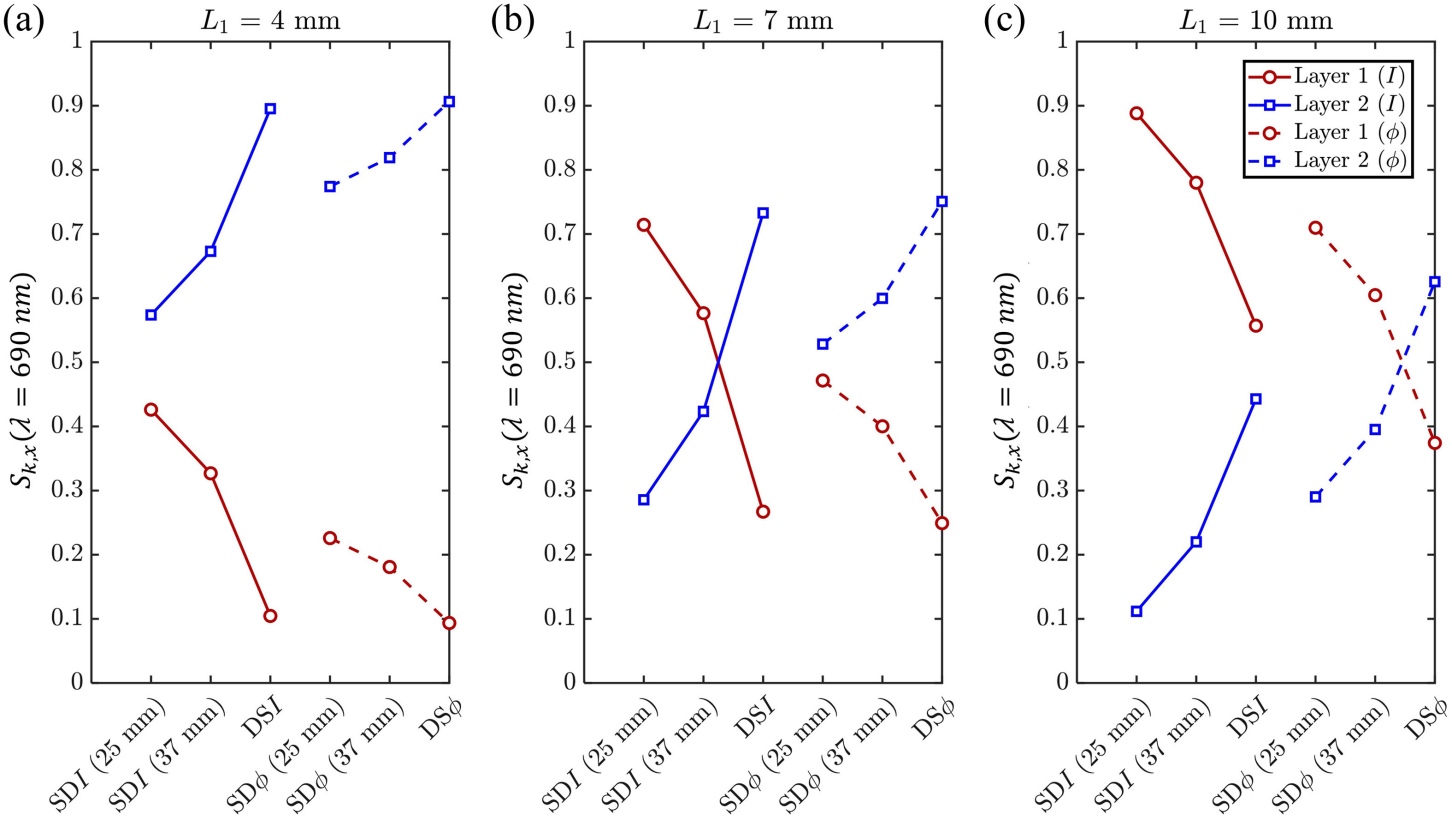
Sensitivity to absorption changes in either layer of a two-layer medium for single-distance intensity (SDI) at 25 and 37 mm, single-distance phase (SDϕ) at 25 and 37 mm, dual-slope intensity (DSI), and dual-slope phase (DSϕ). Each panel corresponds to a different superficial layer thickness: $L_{1}=4\;\mathrm{mm}$ (a), 7 mm (b), and 10 mm (c). Red curves indicate sensitivity to the top layer (layer 1), whereas blue curves indicate sensitivity to the bottom layer (layer 2). Solid lines represent intensity data, and dashed lines represent phase data. The optical properties used to generate these sensitivities with diffusion theory are those reported in Table 2 at a wavelength of 690 nm.

The sensitivity of a given data type to layer 1 (or layer 2) can be expressed in terms of the mean partial generalized pathlength within that layer, relative to the mean total generalized pathlength across all layers. Specifically, the mean partial generalized pathlength for a data type in a given layer is defined as the first derivative of that data type with respect to absorption in that layer. A comprehensive discussion of NIRS sensitivities for CW, TD, and FD modalities is available in a recent review article.^30^ To illustrate these concepts, Fig. 2 shows the sensitivity to absorption changes in either layer of a two-layer medium for various measurement configurations, including SDI at 25 and 37 mm, SDϕ at 25 and 37 mm, DSI, and DSϕ, and for three representative thicknesses of the superficial layer ($L_{1}=4$, 7, and 10 mm). The optical properties used in these simulations are those listed in Table 2 for the 690 nm wavelength. Figure 2 indicates that, in general, the sensitivity to the bottom layer (layer 2) increases as one transitions from data collection in single-distance (at 25 mm), single-distance (at 37 mm), and dual-slope for both intensity and phase. Furthermore, for any data collection configuration (single-distance or dual-slope), phase data feature a greater sensitivity to the bottom layer compared with intensity data.

**Table 2:** Summary of the parameters used in simulations of two-layered and three-layered media, including baseline absorption coefficient ($\mu_{a}$) and baseline reduced scattering coefficient ($\mu_{s}^{\prime }$) at wavelengths of 690 and 830 nm; baseline concentrations of oxygenated hemoglobin (O), deoxygenated hemoglobin (D), and total hemoglobin (T); scattering power; tissue oxygen saturation (${\mathrm{StO}}_{2}$); and water volume fraction.

Layer	$\mu_{a}$ at 690 nm (${\mathrm{mm}}^{-1}$)	$\mu_{a}$ at 830 nm (${\mathrm{mm}}^{-1}$)	$\mu_{s}^{\prime }$ at 690 nm (${\mathrm{mm}}^{-1}$)	$\mu_{s}^{\prime }$ at 830 nm (${\mathrm{mm}}^{-1}$)	O (μM)	D (μM)	T (μM)	Scattering power	${\mathrm{StO}}_{2}$ (%)	Water volume fraction (%)
1	0.0084	0.0110	1	0.98	37.5	12.5	50	0.1	75	20
2	0.0170	0.0233	0.5	0.42	75	25	100	1	75	80
3	0.0084	0.0114	1.5	1.3	37.5	12.5	50	0.7	75	32

For three-layer media, we assumed that $\Delta \mu_{a}$ in the third layer is 0 ($\Delta \mu_{a,3}=0$). Consequently, Eqs. (3) and (4) remain valid for three-layer models. The distinction between the two-layer and three-layer models lies in the sensitivities $S_{k,x}$ and $S_{k,y}$, which differ between two-layer and three-layer models.

## 3 Results

### 3.1 Experimental Results *In Vivo*

#### 3.1.1 Measurement consistency and reproducibility

**Fig. 3:**
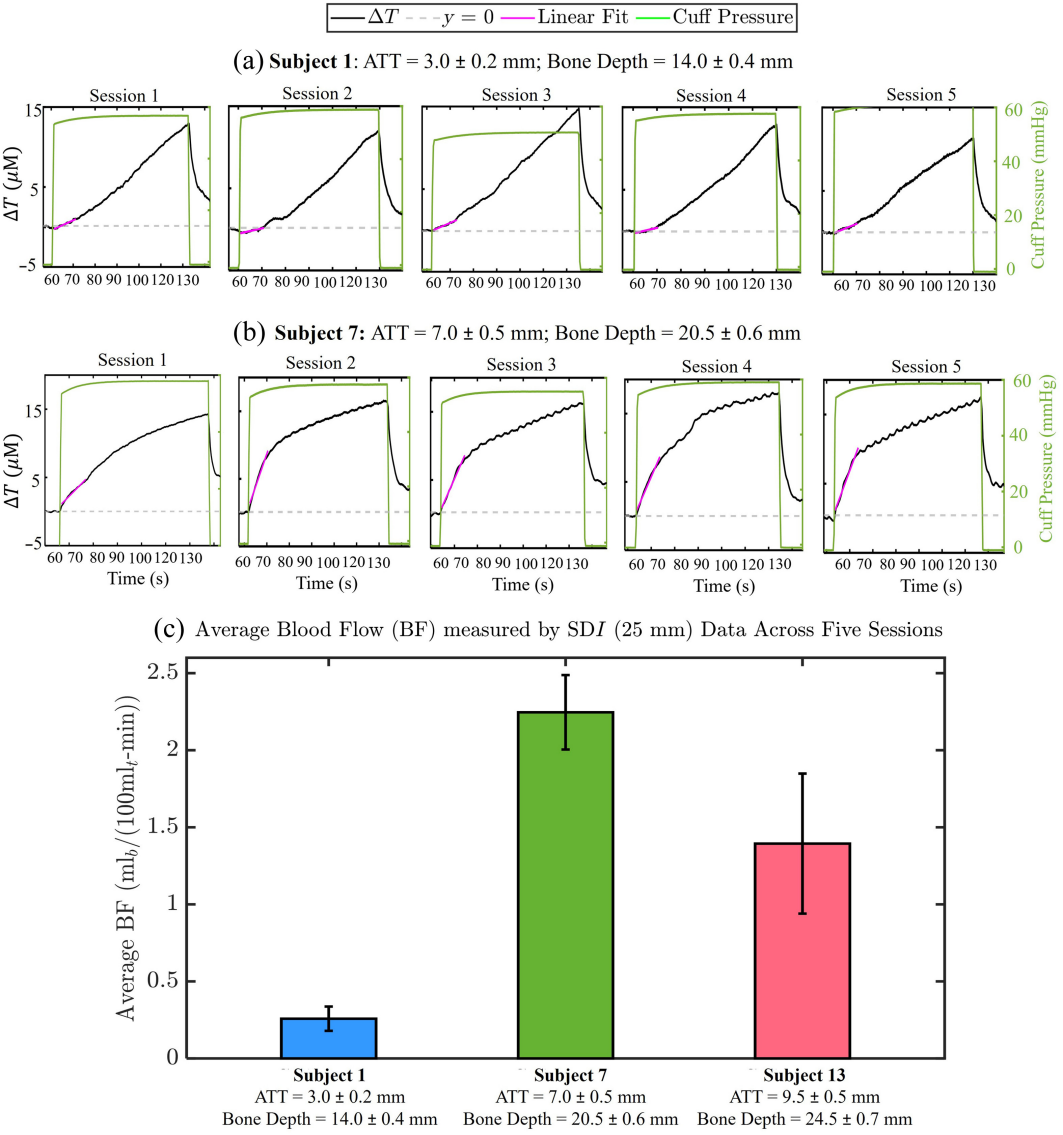
Reproducibility of time traces of ΔT during venous occlusion obtained with SDI measurements at a source–detector distance of 25 mm. (a) Results for subject 1 with a bone depth of 14.0±0.4  mm and ATT of 3.0±0.2  mm across five independent sessions. (b) Results for subject 7 with a bone depth of 20.5±0.6  mm and ATT of 7.0±0.5  mm across five independent sessions. The linear fit over the first 10 s of venous occlusion provides the initial rate of increase in ΔT, used to calculate BF. (c) Mean and standard deviation of BF measured over five independent sessions for subject 1, subject 7, and subject 13 (bone depth = 24.5 ± 0.7 mm; ATT = 9.5 ± 0.5 mm).

To evaluate the consistency and reproducibility of measurements obtained with our newly designed optical probe, we performed a venous occlusion protocol at rest on three subjects across five separate sessions. The subjects included subject 1, with a bone depth of 14.0±0.4  mm and adipose tissue thickness of 3.0±0.2  mm; subject 7, with a bone depth of 20.5±0.6  mm and adipose tissue thickness of 7.0±0.5  mm; and subject 13, with a bone depth of 24.5±0.7  mm and adipose tissue thickness of 9.5±0.5  mm. Figure 3 presents the time traces of ΔT measured with SDI at a source–detector distance of 25 mm for subject 1 [Fig. 3(a)] and subject 7 [Fig. 3(b)] across the five sessions. As shown in Figs. 3(a) and 3(b), there is qualitative reproducibility in both the amplitude and the initial slope of ΔT measurements across sessions. A clear difference is also observed in the initial slopes of ΔT over time, reflecting blood flow differences between the two subjects, with subject 7 exhibiting a significantly steeper slope than subject 1.

Figure 3(c) quantifies the intersession variability of measured blood flow both within individual subjects and across subjects. The average and standard deviation of blood flow measurements across five sessions were as follows: $0.25\pm 0.07\;{\mathrm{mL}}_{\text{blood}}/(100\;{\mathrm{mL}}_{\text{tissue}})/\min$ for subject 1, $2.2\pm 0.2\;{\mathrm{mL}}_{\text{blood}}/(100\;{\mathrm{mL}}_{\text{tissue}})/\min$ for subject 7, and $1.4\pm 0.4{\mathrm{mL}}_{\text{blood}}/(100{\mathrm{mL}}_{\text{tissue}})/\min$ for subject 13. Notably, the standard deviation of repeated blood flow measurements within each subject was relatively small ($\leq 0.4\;{\mathrm{ml}}_{\text{blood}}/(100\;{\mathrm{mL}}_{\text{tissue}})/\min$), highlighting the consistency of the measurements. By contrast, the variation in measured blood flow across subjects was $>1\;{\mathrm{mL}}_{\text{blood}}/(100\;{\mathrm{mL}}_{\text{tissue}})/\min$, reflecting clear physiological differences. These results demonstrate the reliability of our optical probe design in delivering consistent and reproducible blood flow measurements.

#### 3.1.2 Tissue hemodynamics during vascular occlusions

**Fig. 4:**
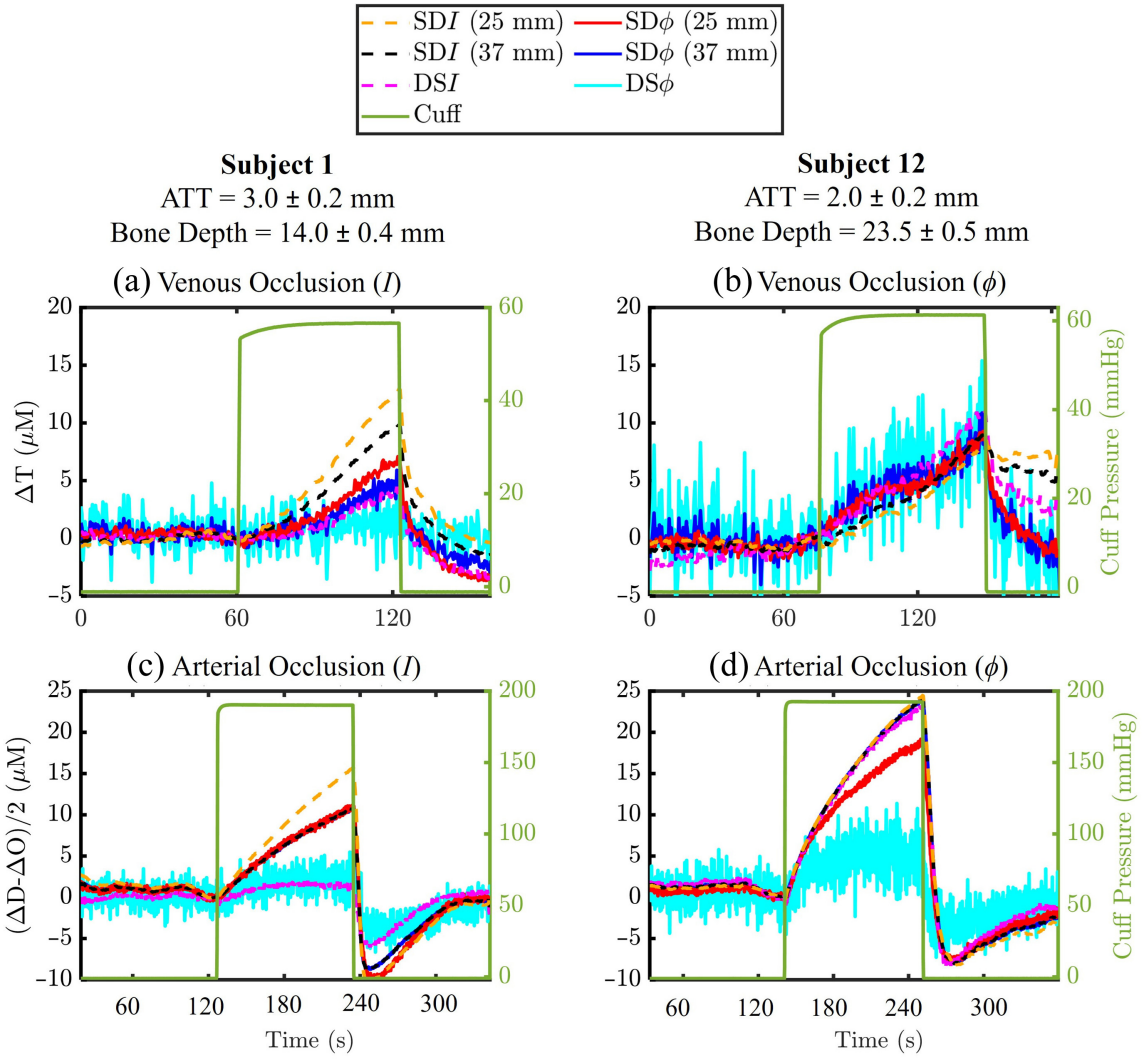
Time traces of changes in hemoglobin concentration during vascular occlusions, obtained with different data types, including SDI (at 25 and 37 mm), SDϕ (at 25 and 37 mm), DSI, and DSϕ. (a) ΔT during venous occlusion derived from intensity (I) data for subject 1 [bone depth = 14.0±0.4  mm; adipose tissue thickness (ATT) = 3.0±0.2  mm]. (b) ΔT during venous occlusion derived from phase (ϕ) data for subject 12 (bone depth = 23.5±0.5  mm; ATT = 2.0±0.2  mm). (c) (ΔD−ΔO)/2 during arterial occlusion derived from intensity (I) data for subject 1. (d) (ΔD−ΔO)/2 during arterial occlusion derived from phase (ϕ) data for subject 12.

To compare tissue hemodynamics among individuals with different anatomical characteristics, we present time traces of changes in chromophore concentrations from venous and arterial occlusion protocols for two representative subjects in Fig. 4. These subjects were selected to illustrate the impact of varying tissue layer thicknesses. Subject 1 was chosen for having a thin adipose tissue layer (3.0±0.2  mm) and the shallowest bone depth (14.0±0.4  mm). By contrast, subject 12 was selected for its thin adipose layer (2.0±0.2  mm), thick bone depth (23.5±0.5  mm), and the greatest muscle thickness (21.5±0.3  mm) among all participants.

Figures 4(a) and 4(b) show time traces of ΔT during venous occlusion for subjects 1 and 12, respectively. Figures 4(c) and 4(d) present time traces of (ΔD−ΔO)/2 during arterial occlusion for the same subjects. The ΔT traces in Fig. 4 were obtained using SDI (at 25 and 37 mm), SDϕ (at 25 and 37 mm), DSI, and DSϕ data types. Phase measurements (SDϕ and DSϕ) generally exhibited higher noise levels compared with intensity measurements (SDI and DSI).

Across all subjects and data types, measured blood flow with the venous occlusion protocol ranged from 0.02 to $2.9\;{\mathrm{mL}}_{\text{blood}}/(100\;{\mathrm{mL}}_{\text{tissue}})/\min$, whereas oxygen consumption with the arterial occlusion protocol ranged from 0.2 to $5.2\;\mu {\mathrm{mol}}_{O2}/(100\;{\mathrm{mL}}_{\text{tissue}})/\min$. These values are comparable to those reported in previous NIRS studies using similar vascular occlusion methods.^16^^,^^31^^,^^32^ The differing values observed for the initial slope of ΔT during venous occlusion and (ΔD−ΔO)/2 during arterial occlusion across the six data types (SDI and SDϕ at 25 and 37 mm, DSI and DSϕ) highlight the varying spatial sensitivities of these data types to inhomogeneous tissue hemodynamics. This observation underscores the importance of considering the layered anatomical structure of tissue when interpreting NIRS data. Measured blood flow and oxygen consumption values for all data types and all subjects are provided in Tables S2 and S3, respectively, of the Supplementary Material. Reported errors reflect the standard deviation of the slopes of the linear fits used to calculate blood flow and oxygen consumption, as described in Eqs. (1) and (2).

Previous NIRS studies investigating blood flow at rest using the same venous occlusion protocol applied here have reported a negligible dependence of blood flow on adipose tissue thickness.^9^ Our earlier FD-NIRS study of forearm blood flow (using a venous occlusion protocol and data analysis based on a two-layer model) found that the *in vivo* results were consistent with comparable blood flow in the top layer (adipose tissue) and bottom layer (muscle tissue).^25^ The potential contribution of bone tissue was not considered in these previous studies. Bone tissue is highly vascularized, and it is estimated that 10% to 15% of the resting cardiac output is directed to the skeleton.^33^ However, we note that the blood flow measurements using FD-NIRS reported in this work reflect a local increase in blood volume that results from vascular dilation or vascular recruitment. The solid nature of compact bone tissue limits changes in bone blood volume^34^ and thus reduces the possibility of an increase in hemoglobin concentration in bone tissue due to venous occlusion. Therefore, we assume a negligible rate of increase of ΔT in bone tissue during venous occlusion (which, within the context of this study, is equivalent to a negligible contribution from bone hemodynamics to the blood flow measurements considered here).

Furthermore, prior NIRS studies of oxygen consumption at rest using the same arterial occlusion protocol employed here have shown a significant decrease in the measured oxygen consumption with increasing adipose tissue thickness, indicating that oxygen consumption in adipose tissue is less than in muscle tissue.^9^^,^^11^^,^^13^^,^^35^ In addition, concurrent measurements of oxygen consumption in the tibia and skeletal muscle at rest with the arterial occlusion method have shown that oxygen consumption in bone tissue is significantly lower than in muscle tissue.^17^^,^^18^^,^^34^

#### 3.1.3 Ratios of measured blood flow and oxygen consumption obtained with different data types

To investigate the spatial distribution of tissue hemodynamics and normalize the individual hemodynamic responses to vascular occlusions, we considered the ratios of blood flow and oxygen consumption obtained with different data types. This approach leverages the varying depth sensitivities of the various FD-NIRS data types. For instance, it is well known that NIRS data collected at longer source–detector distances feature a deeper sensitivity compared with data collected at shorter distances. Accordingly, we first focus on the ratio of blood flow or oxygen consumption obtained from single-distance measurement at a longer source–detector distance at 37 mm relative to those at 25 mm for both intensity and phase data. In this context, a ratio>1 indicates higher blood flow or oxygen consumption in deeper tissues compared with superficial tissues, whereas a ratio<1 suggests lower values in deeper layers. Data from two subjects were excluded from the arterial occlusion analysis due to motion artifacts.

**Fig. 5:**
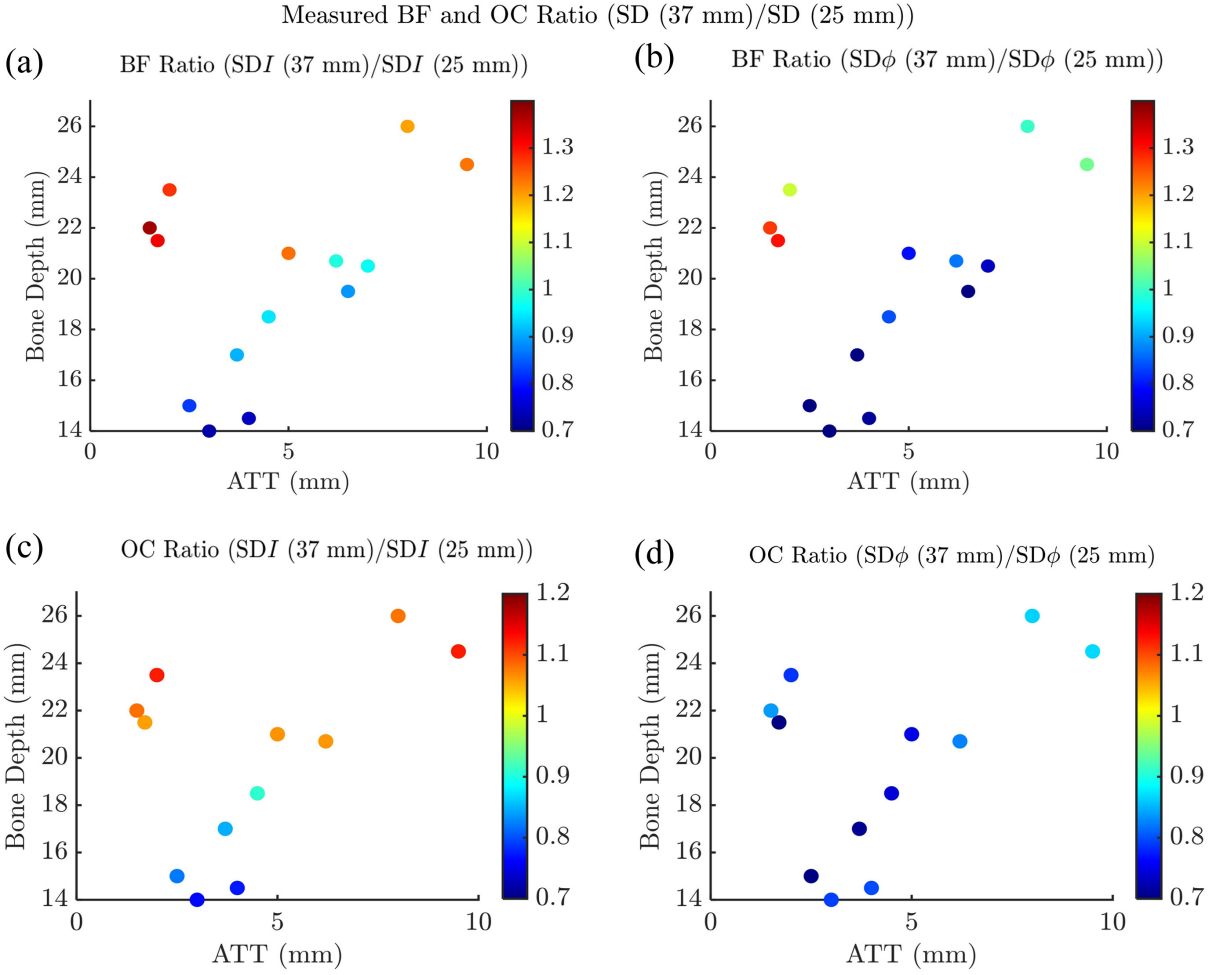
Ratios of measured BF and OC across all subjects, obtained from longer single-distance (SD) at 37 mm relative to those at shorter SD at 25 mm, shown as SD (37 mm)/SD (25 mm), and plotted as a function of bone depth and adipose tissue thickness (ATT). (a) BF ratio for intensity (I) data; (b) BF ratio for phase (ϕ) data; (c) OC ratio for intensity (I) data; (d) OC ratio for phase (ϕ) data.

Figures 5(a) and 5(b) show blood flow ratios from venous occlusion measurements, whereas Figs. 5(c) and 5(d) display oxygen consumption ratios from arterial occlusion measurements. Blood flow and oxygen consumption ratios for both intensity and phase data tend to increase with bone depth, suggesting that contributions from the bone tissue lead to lower measured values of blood flow and oxygen consumption. Subjects with thinner adipose tissue and thicker muscle layers generally exhibited higher blood flow and oxygen consumption ratios, reflecting greater blood flow and oxygen consumption in muscle compared with adipose tissue. For instance, subject 11 (bone depth=22.0±0.6  mm, adipose thickness=1.5±0.1  mm, and muscle thickness=20.5±0.5  mm) showed blood flow ratios>1 for both intensity and phase measurements.

Phase measurements consistently yielded lower blood flow and oxygen consumption ratios than intensity measurements, with oxygen consumption ratios from phase data that were <1 in all subjects. This result indicates a higher sensitivity to deeper bone tissue, where blood flow and oxygen consumption are comparatively lower.

Our previous work demonstrated that the dual-slope configuration provides increased sensitivity to deeper tissues compared with a single-distance configuration.^23^^,^^24^ Building on this, we analyzed the ratio of blood flow obtained from dual-slope data relative to that from shorter single-distance measurements at 25 mm for both intensity and phase data types. Figures S1(a)–S1(f) in the Supplementary Material show the measured blood flow ratios, whereas Figs. S1(g)–S1(l) show oxygen consumption ratios. The results in Fig. S1 are consistent with those reported in Fig. 5, reaffirming that dual-slope measurements, similar to single-distance measurements at 37 mm, exhibit greater sensitivity to deeper tissue than single-distance measurements at 25 mm. Notably, for subjects with shallower bone depths, both blood flow and oxygen consumption ratios obtained from the dual-slope over single-distance at 25 mm are lower as shown in Fig. S1, in some cases even approaching 0, relative to the corresponding ratios obtained from single-distance at 37 mm over single-distance at 25 mm measurements, as shown in Fig. 5. Considering that bone tissue features lower blood flow (or, more precisely, a smaller rate of increase of blood volume during venous occlusion) and a lower oxygen consumption than muscle tissue, this observation suggests that dual-slope data (collected at 25 and 37 mm source–detector distances) are more sensitive to deeper bone tissue than single-distance data collected at 37 mm. Conversely, in cases where the bone is deeper and does not offer significant contributions to the measured signal, higher blood flow and oxygen consumption ratios are observed for dual-slope over single-distance at 25 mm measurements than for single-distance at 37 mm over single-distance at 25 mm measurements. This indicates a reduced sensitivity of dual-slope versus single-distance at 37 mm data to the superficial adipose tissue, which also has lower blood flow and oxygen consumption than muscle tissue. In addition, consistent with the results reported in Fig. 5, blood flow and oxygen consumption ratios derived from phase data are lower than those from intensity data. This confirms the enhanced sensitivity to deeper tissues offered by phase measurements and underscores the utility of combining phase and intensity data to improve the assessment of heterogeneous tissue hemodynamics.

### 3.2 Theoretical Simulations for Two-Layered and Three-Layered Media

We conducted theoretical simulations based on diffusion theory for two-layer and three-layer media to guide the interpretation of the *in vivo* results from both occlusion protocols. Table 2 summarizes all the simulation parameters, including baseline optical properties, hemoglobin concentrations, scattering power (i.e., the exponent b of the scattering wavelength dependence $\lambda^{-b}$), tissue oxygen saturation (${\mathrm{StO}}_{2}$), and water content in each layer. In these simulations, layer 1 represented adipose tissue, layer 2 represented muscle tissue, and layer 3 represented bone tissue. For the two-layer simulations, only adipose and muscle tissues (layers 1 and 2) were modeled, whereas the three-layer simulations, in addition, included bone tissue as layer 3.

The optical properties of adipose and muscle tissues, with a higher $\mu_{s}^{\prime }$ and lower $\mu_{a}$ in adipose tissue compared with muscle tissue, were chosen based on previously reported values for lipids and muscle tissue,^36^^,^^37^ as well as our previous findings.^21^ For adipose tissue (layer 1), baseline optical parameters were set as follows: $\mu_{a}$ of $0.0083\;{\mathrm{mm}}^{-1}$ at 690 nm and $0.0104\;{\mathrm{mm}}^{-1}$ at 830 nm, corresponding to a baseline T of 50  μM, an ${\mathrm{StO}}_{2}$ of 75%, and a water volume fraction to 20%.^38^ $\mu_{s}^{\prime }$ was set to $1.0\;{\mathrm{mm}}^{-1}$ at 690 nm and $0.98\;{\mathrm{mm}}^{-1}$ at 830 nm, using a scattering power of 0.1.^39^

For muscle tissue (layer 2), baseline $\mu_{a}$ was set to $0.0166\;{\mathrm{mm}}^{-1}$ at 690 nm and $0.0208\;{\mathrm{mm}}^{-1}$ at 830 nm, corresponding to a baseline T of 100  μM (with a blood volume fraction of 4.3%), an ${\mathrm{StO}}_{2}$ of 75% (assuming no difference between ${\mathrm{StO}}_{2}$ in adipose and muscle tissues),^35^ and a water volume fraction of 80%.^40^ $\mu_{s}^{\prime }$ was set to $0.5\;{\mathrm{mm}}^{-1}$ at 690 nm and $0.42\;{\mathrm{mm}}^{-1}$ at 830 nm, using a scattering power of 1.^39^

For the three-layer simulations, bone tissue (layer 3) was modeled with a baseline $\mu_{a}$ of $0.0083\;{\mathrm{mm}}^{-1}$ at 690 nm and $0.0104\;{\mathrm{mm}}^{-1}$ at 830 nm, corresponding to a baseline T of 50  μM (with a blood volume fraction of 2.2%),^41^ an ${\mathrm{StO}}_{2}$ of 75%, and a water volume fraction of 32%.^40^ $\mu_{s}^{\prime }$ was set to $1.5\;{\mathrm{mm}}^{-1}$ at 690 nm and $1.3\;{\mathrm{mm}}^{-1}$ at 830 nm, using a scattering power of 0.7.^42^ In all layers, the $\mu_{a}$ values at 690 and 830 nm were determined using Beer’s law, calculated from the respective baseline hemoglobin concentrations, ${\mathrm{StO}}_{2}$, and water content, incorporating the extinction coefficients of oxyhemoglobin and deoxyhemoglobin, as well as the absorption coefficient of water.

#### 3.2.1 Simulations of hemodynamics associated with venous occlusion

A key observation from our *in vivo* measurements during venous occlusion (see Fig. 5) is that blood flow ratios increase with bone depth, transitioning from values <1 for bone depths <20  mm to greater values (>1 in the case of intensity measurements and ∼1 for phase measurements) when bone depth exceeds 20 mm. The objective of the theoretical simulations was to replicate these trends and to identify the conditions that are consistent with these trends observed *in vivo*. To simulate the layer-specific increase in ΔT during venous occlusion, we assigned values of $\Delta T_{k}$ in the k’th layer, where $\Delta T_{k}$ is assumed proportional to blood flow in that layer and reflects blood accumulation. This proportionality ensures that the blood flow ratios reported in Fig. 5 correspond to the ΔT ratios derived from the simulations using Eq. (3).

To explore the effects of differential blood flow between adipose and muscle tissues, we fixed the change in total hemoglobin concentration in the second layer (muscle) ($\Delta T_{2}$) to 10  μM while varying the change in total hemoglobin concentration of the first layer (adipose tissue) ($\Delta T_{1}$) from 2 to 20  μM. This range represents scenarios of lower, equal, or higher blood flow in adipose tissue relative to muscle. To account for negligible blood flow in bone tissue (i.e., negligible increase in blood volume), the change in total hemoglobin concentration in the third layer ($\Delta T_{3}$) was set to 0 in the three-layer simulations.

Figures S2(a) and S2(b) in the Supplementary Material show the results of two-layer simulations of blood flow measured at a longer single-distance at 37 mm divided by the blood flow measured at a shorter single-distance at 25 mm for both intensity (SDI) and phase (SDϕ), plotted as a function of $L_{1}$ (adipose tissue thickness). Figures S2(c) and S2(d) in the Supplementary Material present the corresponding blood flow ratios from three-layer simulations as a function of $L_{1\;}+L_{2}$ (bone depth), with $L_{1}$ set to 1.5 mm (qualitatively similar results were observed for $L_{1}=1.5$, 5, and 9.5 mm). As shown in Figs. S2(a) and S2(b) in the Supplementary Material, simulations for the two-layer medium, which neglect bone tissue, failed to reproduce the transition of blood flow ratios from values <1 to >1 (for intensity) or ∼1 (for phase) as observed *in vivo* (see Fig. 5). By contrast, this transition was successfully replicated in the three-layer simulations [see Figs. S2(c) and S2(d) in the Supplementary Material] that account for the impact of bone tissue as the third tissue layer. Furthermore, the three-layer simulations indicate that resting blood flow in adipose tissue is lower than in muscle tissue as only simulations with $\Delta T_{1}<\Delta T_{2}$ reproduced the transition from blood flow ratios <1 to >1 as a function of bone depth that is observed in the intensity measurements [see Fig. S2(c) in the Supplementary Material]. The three-layer simulations also found that blood flow ratios derived from phase measurements are consistently lower than those obtained from intensity measurements, in good agreement with the *in vivo* results [see Fig. S2(d) in the Supplementary Material], though they consistently remained <1, whereas *in vivo* data occasionally exhibited ratios >1. These simulation results provide strong evidence that bone tissue, as represented by the third layer in the three-layer model, contributed to the *in vivo* measurements, particularly at shallower bone depths (<20  mm).

#### 3.2.2 Simulations of hemodynamics associated with arterial occlusion

To simulate the layered hemoglobin desaturation during arterial occlusion and assess the impact of varying relative oxygen consumption in adipose, muscle, and bone tissues, we modeled ΔO and ΔD in each tissue layer. Specifically, we fixed the change in deoxyhemoglobin concentration in the second layer (muscle tissue) ($\Delta D_{2}$) to 20  μM and set $\Delta O_{2}=-\Delta D_{2}=-20\;\mu M$, representing hemoglobin desaturation with no change in total hemoglobin concentration. The change in deoxyhemoglobin concentration in the first layer (adipose tissue) ($\Delta D_{1}$) varied from 2 to 24  μM, with corresponding $\Delta O_{1}$ values ranging from −2 to −24  μM, to simulate scenarios where oxygen consumption in adipose tissue is lower than, equal to, or greater than in muscle tissue. To account for the lower oxygen consumption in bone tissue, both $\Delta D_{3}$ and $\Delta O_{3}$ were set to 0 in the three-layer simulations. In this framework, $\Delta D_{k}$ is proportional to oxygen consumption within the k’th layer. This proportionality ensures that the oxygen consumption ratios reported in Fig. 5 (for single-distance measurements) and Fig. S1 in the Supplementary Material (for dual-slope measurements) correspond directly to the (ΔD−ΔO) ratios derived from the simulations, as defined in Eq. (4).

Results from the two- and three-layer simulations for oxygen consumption ratios closely mirrored those observed for blood flow ratios in Fig. S2 in the Supplementary Material and are therefore not reported here. As with the blood flow measurements, the two-layer simulations failed to reproduce the trends of oxygen consumption ratios observed *in vivo*, whereas the three-layer simulations successfully reproduced these trends, as long as $\Delta D_{2}>\Delta D_{1}$. This outcome supports the observation that oxygen consumption in muscle tissue exceeds that in adipose tissue.

#### 3.2.3 Simulations of hemodynamics associated with varying tissue thicknesses

To further interpret the *in vivo* results, we performed simulations using both two-layer and three-layer models, adopting values of $L_{1}$ (adipose tissue thickness) and $L_{1\;}+L_{2}$ (bone depth) that span the full range of values reported in Table 1 for the human subjects enrolled in this study. These simulations were used to compute blood flow and oxygen consumption ratios from measurements at short (25 mm) and long (37 mm) source–detector separations for both intensity and phase data, as a function of $L_{1}$ (adipose tissue thickness) and $L_{1}+L_{2}$ (bone depth). In this limiting case, we observe that the blood flow and oxygen consumption ratios take values that approach 1 for small $L_{1}$ (when both short- and long-distance data mostly probe muscle tissue) and for large $L_{1}$ (when both short- and long-distance data mostly probe adipose tissue). The blood flow and oxygen consumption ratios take values > 1 at intermediate values of $L_{1}$, when the long-distance data are more strongly impacted by muscle tissue than the short-distance data. It is noteworthy that the value of $L_{1}$ where the ratios are maximal is greater for phase data than intensity data, which is a further indication of the deeper sensitivity of phase versus intensity data.

The three-layer simulations provide a more general description of the relative contributions of adipose, muscle, and bone tissues to the blood flow and oxygen consumption ratios. Specifically, for a given adipose tissue thickness, blood flow and oxygen consumption ratios tend to increase as a function of bone depth (because of the increased contribution of muscle tissue to the long-distance measurements), whereas for a given bone depth, the ratios first increase as a function of adipose tissue thickness (because of the increased contribution of adipose tissue to the short-distance measurements) and then decrease toward a value of 1 (because of the dominant impact of adipose tissue on both short- and long-distance measurements). Notably, oxygen consumption ratios obtained from phase data were consistently lower than those from intensity data, and the simulated oxygen consumption ratios in the three-layer model were also consistently lower than the corresponding blood flow ratios.

**Fig. 6:**
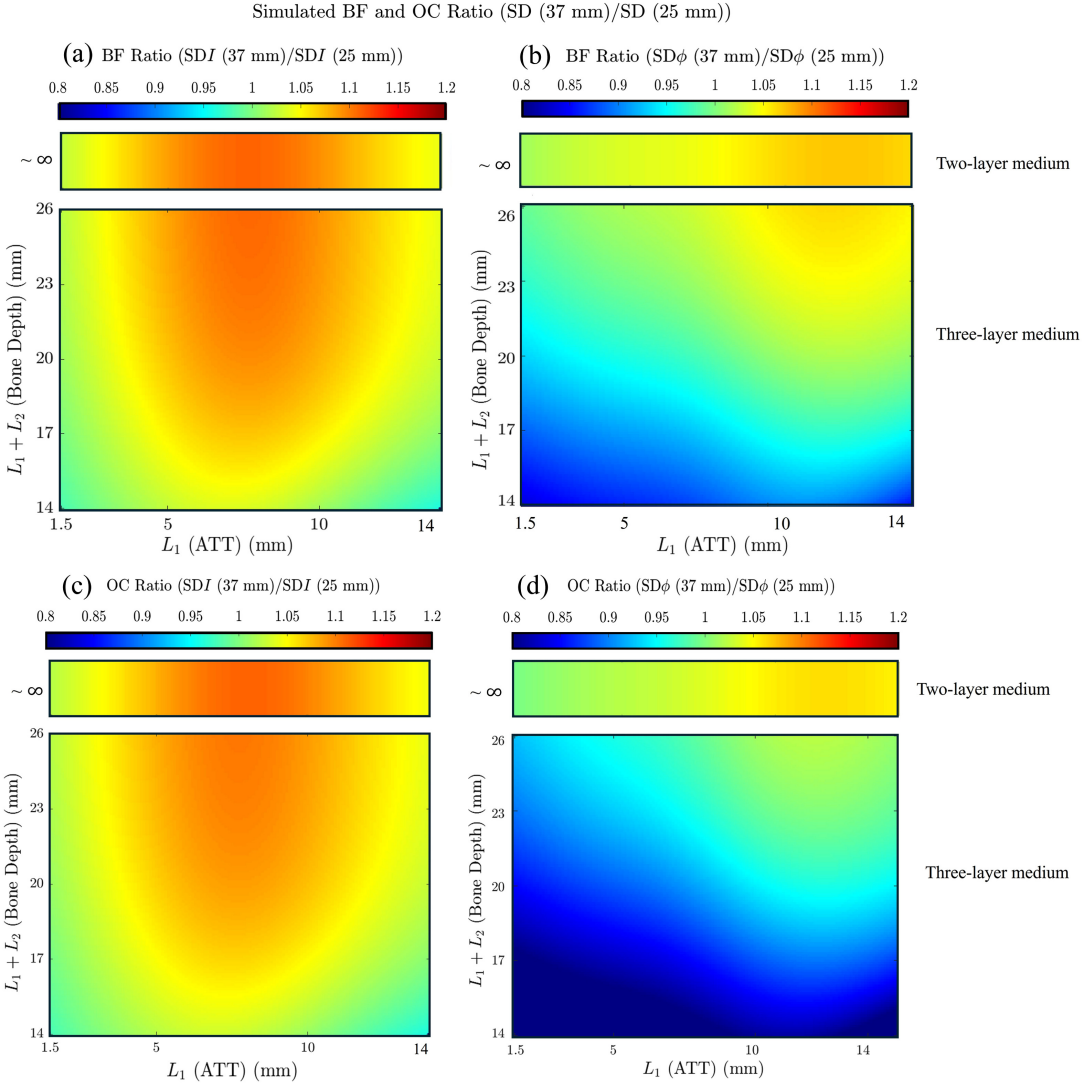
Ratios of simulated BF and OC obtained from longer single-distance (SD) at 37 mm relative to those at shorter SD at 25 mm, shown as SD (37 mm)/SD (25 mm), and plotted as functions of adipose tissue thickness ($L_{1}$) and bone depth ($L_{1\;}+L_{2}$). (a) BF ratio for intensity (I) data; (b) BF ratio for phase (ϕ) data; (c) OC ratio for intensity (I) data; (d) OC ratio for phase (ϕ) data. Each subfigure shows results for both a two-layer model (for which $L_{1}+L_{2}∼\infty$) and a three-layer model. For blood flow simulations, $\Delta T_{1}$, $\Delta T_{2}$, and $\Delta T_{3}$ were set to 5, 10, and 0  μM, respectively. For oxygen consumption simulations, $\Delta D_{1}$, $\Delta D_{3}$, and $\Delta D_{3}$ were set to 5, 10, and 0  μM, respectively. For all simulations, $\mu_{a}$ and $\mu_{s}^{\prime }$ for each layer (layers 1, 2, and 3, respectively) were set to $\mu_{a}=(0.0083,0.0166,\;\text{and}\;0.0083)\;{\mathrm{mm}}^{-1}$ at λ=690  nm, $\mu_{a}=(0.0104,0.0208,\;\text{and}\;0.0104)\;{\mathrm{mm}}^{-1}$ at λ=830  nm, $\mu_{s}^{\prime }=(1,0.5,\;\text{and}\;1.5)\;{\mathrm{mm}}^{-1}$ at λ=690  nm, and $\mu_{s}^{\prime }=(0.98,0.42,\;\text{and}\;1.3)\;{\mathrm{mm}}^{-1}$ at λ=830  nm (see Table 2).

Considering the deeper sensitivity of dual-slope measurements compared with single-distance measurements, we also performed simulations of blood flow and oxygen consumption ratios obtained from dual-slope data relative to single-distance data at 25 mm, as considered in the experimental results of Fig. S1 in the Supplementary Material. The results of these simulations are shown in Fig. S3 in the Supplementary Material. They are qualitatively similar to the results of the simulations presented in Fig. 6, but in a comparison with the ratios reported in Fig. 6, we note larger deviations from 1 (both for the values <1 at small $L_{1}$ and for those >1 at intermediate values of $L_{1}$) in the ratios obtained from intensity data and the shift to greater values of $L_{1}$ for the maximum ratios obtained from phase data. These indications point, again, to the deeper sensitivity of dual-slope data (both intensity and phase) relative to the corresponding single-distance data. It is striking how the three-layer simulations of Figs. 6 and S3 in the Supplementary Material, as discussed in this section, reproduce the salient features of the *in vivo* results of Figs. 5 and S1 in the Supplementary Material.

#### 3.2.4 Simulations of varying baseline optical properties in each tissue layer

To assess the influence of baseline optical properties on blood flow and oxygen consumption measurements, we performed additional theoretical simulations varying baseline $\mu_{s}^{\prime }$ and $\mu_{a}$ of each layer within biologically relevant ranges. This analysis focused on how different optical properties in each layer affect the results and interpretation of FD-NIRS signals.

For scattering properties, simulations were conducted with the reduced scattering coefficient of layer 1 (adipose tissue) ($\mu_{s,1}^{\prime }$) ranging from 0.3 to $1.5\;{\mathrm{mm}}^{-1}$, encompassing values below, equal to, and above the value of reduced scattering coefficient in layer 2 (muscle tissue) ($\mu_{s,2}^{\prime }$), which was fixed at $0.5\;{\mathrm{mm}}^{-1}$. All other parameters were kept constant, as listed in Table 2. Results showed that the *in vivo* trends were reproduced when $\mu_{s,1}^{\prime }>\mu_{s,2}^{\prime }$. By contrast, when $\mu_{s,1}^{\prime }\leq \mu_{s,2}^{\prime }$, ratios fell below 1 across all measurement configurations, which is inconsistent with *in vivo* results. This reinforces previous findings that adipose tissue typically has a greater $\mu_{s}^{\prime }$ than muscle tissue^25^.

For absorption properties, simulations were performed by varying the absorption coefficient of layer 1 ($\mu_{a,1}$) between 0.003 and $0.029\;{\mathrm{mm}}^{-1}$, spanning values lower, equal to, and higher than that of layer 2 ($\mu_{a,2}=0.018\;{\mathrm{mm}}^{-1}$). Unlike scattering properties, varying the absorption coefficient of the top layer had a negligible influence on the simulation results. This suggests that the baseline absorption characteristics of the superficial layer have minimal impact on the measurement depth sensitivity or the resulting hemodynamic changes. This observation is consistent with the similar $\mu_{a}$ values reported for adipose and muscle tissues in our previous work.^25^

## 4 Discussion

The new optical probe developed for this study features pneumatic actuators to consistently place optodes in contact with the tissue with a controllable pressure, allowing for reproducible measurements *in vivo*. It also minimally impacts tissue hemodynamics by securing contact of the optical fibers onto the skin with minimal pressure and by obviating the need to wrap bandages around the arm to firmly secure the optical probe. Therefore, the probe design was extremely valuable in this study aimed at the analysis of the impact of bone tissue and adipose tissue in NIRS measurements of the brachioradialis muscle. However, this probe design is only suitable for a stationary subject and may be cumbersome for use on other muscles (gastrocnemius, etc.), so modification may be needed for broader applicability to muscle studies.

The primary takeaway of this study is that, in addition to superficial adipose tissue, deeper bone tissue may also contribute to the optical NIRS signals measured in noninvasive muscle studies. Similar to the importance of accounting for the contributions of adipose tissue, which has received significant attention in the field, potential bone tissue contributions may also confound measurements of muscle hemodynamics. As expected, the results reported in this study have shown that the impact of bone tissue depends on bone depth in relation to the source–detector distance used for NIRS measurements. Furthermore, we have also shown that the impact of bone tissue depends on the data type (intensity versus phase measurements in FD-NIRS) and data collection mode (single-distance versus multidistance/dual-slope). Although the reported results have indicated that the contributions from deep bone tissue are greater for data collected at longer versus shorter source–detector distances, for phase versus intensity measurements, and for dual-slope versus single-distance measurements, it has not provided quantitative indications of the extent of the bone tissue contributions at a given tissue depth. Indeed, the results of this study do not lead to the discrimination of the contributions from bone and adipose tissues as both tend to decrease the measured hemoglobin accumulation rate and the hemoglobin desaturation rate that are induced by venous occlusion and arterial occlusion, respectively.

With the above caveats, an examination of Figs. 5 and 6 allows the determination that bone tissue at a depth less than ∼20  mm has a significant impact on FD-NIRS data collected at a single-distance measurement at 37 mm (and, from an examination of Figs. S1 and S3 in the Supplementary Material, an even greater impact on dual-slope measurement collected at 25 and 37 mm). From this observation, it follows that to minimize contributions from deep bone tissue, it is important to collect NIRS data at a source–detector distance that does not exceed ∼1.5 times the bone depth. Although this is a rough estimate that depends on anatomical details, the individual optical properties of adipose and muscle tissues, the data type (intensity or phase), and the data collection configuration (single-distance or dual-slope), it nevertheless supports a recommendation based on this study for the future design of NIRS instrumentation for muscle studies. The recommendation is to collect optical data that provide a range of depth sensitivities to allow for the selection of optimal muscle sensitivity after taking into consideration the adipose tissue thickness and bone depth. This goal may be achieved using optical probes with a range of source–detector separations and/or time-resolved methods to provide access to data types with individual spatial regions of sensitivity (amplitude and phase in FD-NIRS, moments of the time-of-flight distribution in TD NIRS, etc.). Moreover, multidistance methods, or dual-slope methods such as the ones applied in this study, also allow for the collection of data with individual and complementary regions of sensitivity. Moreover, we have used only one dual-slope set in this work, based on data at two source–detector separations of 25 and 37 mm. Expanding the probe design to include multiple dual-slope sets and a broader range of source–detector separations would enable the use of a two-layer inversion model, potentially allowing for the discrimination between adipose and muscle tissue hemodynamics.

Another point of discussion is that the results of the three-layered medium simulations showed that the reported *in vivo* data are consistent with smaller blood flow and oxygen consumption in adipose tissue than in muscle tissue at rest. Although a smaller oxygen consumption in adipose versus muscle tissues (at rest) was previously reported using NIRS and arterial occlusion,^9^^,^^11^^,^^13^^,^^35^ blood flow measurements with NIRS and venous occlusion were found to be either poorly correlated with adipose tissue thickness^9^ or consistent with a comparable (or smaller) blood flow in adipose versus muscle tissues on the basis of two-layer theoretical simulations.^25^ These previous NIRS studies,^9^^,^^25^ which were both conducted on the human forearm using source–detector distances of 25 and 35 mm, did not consider the potential impact of bone tissue in their measurements. The lack of correlation of blood flow measurements with adipose tissue thickness may not necessarily reflect a comparable blood flow in adipose and muscle tissues but may also result from bone tissue contributions (which tend to decrease the measured blood flow) at low values of adipose tissue thickness (if concurrent with small values of bone depth). Similarly, theoretical simulations based on two-layer media cannot take into account contributions from bone tissue (which is modeled by the third layer in the three-layer simulations reported here). A lower blood flow in subcutaneous fat than in skeletal muscle is consistent with the fact that the majority of total forearm blood flow is associated with skeletal muscle, with less than 20% being associated with subcutaneous adipose tissue.^43^ Finally, positron emission tomography studies have reported that blood flow in femoral skeletal muscle is significantly greater than in the overlying subcutaneous fat in human subjects.^44^^,^^45^

A modeling assumption in this study is that the layered media considered in the theoretical simulations have an infinite lateral extent. Although this assumption does not fully capture the finite lateral dimensions and curvature of anatomical structures, we observe that the objective of this study is not to investigate the quantitative contributions of bone tissue to the optical measurements but rather to qualitatively compare the relative sensitivities of the different FD-NIRS data types considered here (intensity or phase collected in single-distance or dual-slope configurations) to adipose, muscle, and bone tissue. For such a comparison, the layered geometry allows for the analytical modeling of relative contributions from tissues at different depths (most superficial adipose tissue, deeper muscle tissue, and even deeper bone tissue). Furthermore, the analysis presented here, based on ratios of hemodynamic changes measured with different data types, further reduces the impact of the anatomical details of the tissue considered. Of course, finite-element methods or Monte Carlo simulations can advance the quantitative aspects of this analysis by incorporating anatomically realistic geometries and further refining the analysis of relative contributions from adipose, muscle, and bone tissues to FD-NIRS data.

## 5 Conclusion

This study demonstrates that bone tissue may contribute to noninvasive optical measurements of skeletal muscle hemodynamics. Using a custom-built optical probe with source–detector distances of 25 and 37 mm, our *in vivo* results obtained with different data types (intensity versus phase and single-distance versus dual-slope) revealed that bone depths less than ∼20  mm influence blood flow and oxygen consumption measurements. These findings were further supported by diffusion theory simulations using two-layer and three-layer models, which confirmed the possibility of noticeable contributions from bone tissue. Consequently, in addition to the well-established effects of adipose tissue, the influence of bone tissue must be carefully considered in noninvasive optical measurements of skeletal muscle hemodynamics.

Future research should aim to advance FD-NIRS methodologies for skeletal muscle assessment by optimizing the selection of source–detector distances and refining measurement approaches to enhance sensitivity to muscle tissue while minimizing contributions from adipose and bone tissues. Validation studies involving larger and more diverse populations, including subjects with varying adipose tissue thickness and bone depths, will be critical to identify optimal practices for improving the accuracy and robustness of FD-NIRS measurements of skeletal muscle. Furthermore, data collection using multidistance approaches should be leveraged to minimize contributions from nontarget tissues, such as adipose tissue and bone while enhancing the specificity to muscle tissue. These advancements will facilitate more reliable noninvasive optical measurements of skeletal muscle, enabling improved investigations of functional, physiological, and pathological conditions.

## Supplementary Material

10.1117/1.BIOS.2.3.035002.s01

## Data Availability

Data and code to replicate the figures supporting the major findings of this study are publicly accessible through the DOIT Lab Dataverse repository hosted by Tufts University at https://doi.org/10.7910/DVN/3GXAKA.
