# Supplementary material for: Noninvasive measurements of skeletal muscle hemodynamics using frequency-domain near-infrared spectroscopy: contributions from adipose and bone tissues
Source: Biophotonics Discov. 2025 Sep 17;2(3):035002. doi: 10.1117/1.BIOS.2.3.035002 (PMC13052493; doi:10.1117/1.BIOS.2.3.035002)
Supplement: Supplementary file 1 [file BIOS_002_035002_SD001.pdf]

Supplementary Material

Table S1 Summary of baseline optical properties, including absorption coefficient (μ_a) and reduced scattering coefficient (μ_s') during venous occlusion (VO) and arterial occlusion (AO) and at two wavelengths (λ) of 690 and 830 nm. Optical properties are reported as mean ± standard deviation during baseline, at rest, before occlusions. We note that the errors reported in Table S1 for μ_a and μ_s' reflect the precision of the measured optical properties at each data point, estimated by the standard deviation of the measurements during the baseline period. We further note that, although the reduced scattering coefficient μ_s' is generally expected to decrease as wavelength increases, we observed cases where μ_s' at 830 nm was greater than at 690 nm. This anomalous scattering behavior may result from the fact that the μ_a and μ_s' values reported in Table S1 are effective values obtained under the assumption of tissue homogeneity. In fact, we have observed that by generating diffuse reflectance data using diffusion theory for a two-layer medium and then obtaining effective μ_a and μ_s' by inverting these simulated data with a homogeneous, semi-infinite diffusion model, it is possible for the effective μ_s' to increase with wavelength even though the actual μ_s' of both layers decreases with wavelength. Such occurrence depends on the specific optical properties of the two layers and on the thickness of the top layer, and of course it may also be impacted by the details of spatial tissue heterogeneity (two-layered, three-layered, multi-layered, etc.). N/A indicates data not applicable for subjects whose data were excluded due to motion artifacts during arterial occlusion.

	**Baseline μ_a (mm^{-1})**				**Baseline μ_s' (mm^{-1})**			
**No.**	VO		AO		VO		AO	
	λ = 690 nm	λ = 830 nm	λ = 690 nm	λ = 830 nm	λ = 690 nm	λ = 830 nm	λ = 690 nm	λ = 830 nm
**1**	0.0163 ± 0.0002	0.0140 ± 0.0002	0.0129 ± 0.0001	0.0156 ± 0.0002	0.437 ± 0.002	0.400 ± 0.003	0.411 ± 0.004	0.373 ± 0.002
**2**	0.0259 ± 0.0001	0.0123 ± 0.0001	0.0116 ± 0.0001	0.0120 ± 0.0001	0.862 ± 0.005	0.440 ± 0.002	0.497 ± 0.008	0.461 ± 0.005
**3**	0.0234 ± 0.0001	0.0198 ± 0.0003	0.0245 ± 0.0005	0.0213 ± 0.0004	0.41 ± 0.02	0.376 ± 0.004	0.409 ± 0.008	0.373 ± 0.005
**4**	0.0196 ± 0.0003	0.0167 ± 0.0001	0.0139 ± 0.0002	0.0101 ± 0.0001	0.400 ± 0.001	0.385 ± 0.002	0.336 ± 0.003	0.320 ± 0.002
**5**	0.0211 ± 0.0004	0.0167 ± 0.0002	0.0239 ± 0.0005	0.0181 ± 0.008	0.412 ± 0.008	0.461 ± 0.005	0.44 ± 0.01	0.433 ± 0.006
**6**	0.0163 ± 0.0001	0.0129 ± 0.0001	N/A	N/A	0.384 ± 0.002	0.459 ± 0.003	N/A	N/A
**7**	0.0091 ± 0.0001	0.0094 ± 0.0001	N/A	N/A	0.460 ± 0.002	0.447 ± 0.001	N/A	N/A
**8**	0.0193 ± 0.0002	0.0162 ± 0.0002	0.0188 ± 0.0002	0.0164 ± 0.0002	0.414 ± 0.004	0.485 ± 0.004	0.410 ± 0.003	0.488 ± 0.003
**9**	0.0189 ± 0.0003	0.0165 ± 0.0002	0.0188 ± 0.0003	0.0165 ± 0.0001	0.355 ± 0.005	0.442 ± 0.005	0.364 ± 0.005	0.453 ± 0.002
**10**	0.0252 ± 0.0005	0.0195 ± 0.0003	0.0176 ± 0.0003	0.0213 ± 0.0004	0.372 ± 0.008	0.422 ± 0.005	0.474 ± 0.007	0.273 ± 0.005
**11**	0.0274 ± 0.0004	0.0176 ± 0.0002	0.0224 ± 0.0001	0.0173 ± 0.0002	0.260 ± 0.005	0.323 ± 0.002	0.273 ± 0.004	0.323 ± 0.002
**12**	0.0180 ± 0.0001	0.0204 ± 0.0002	0.0177 ± 0.0002	0.0184 ± 0.0001	0.426 ± 0.003	0.245 ± 0.003	0.430 ± 0.004	0.373 ± 0.002
**13**	0.0086 ± 0.0001	0.0112 ± 0.0001	0.0097 ± 0.0001	0.0104 ± 0.0001	0.530 ± 0.002	0.514 ± 0.003	0.497 ± 0.002	0.467 ± 0.003
**14**	0.0087 ± 0.0001	0.0090 ± 0.0001	0.0082 ± 0.0001	0.0088 ± 0.0001	0.478 ± 0.002	0.437 ± 0.001	0.473 ± 0.002	0.439 ± 0.002

Table S2 Summary of the measured blood flow (BF) obtained from single-distance intensity (SD*I*) at 25 and 37 mm, dual-slope intensity (DS*I*), single-distance phase (SDϕ) at 25 and 37 mm, and dual-slope phase (DSϕ), respectively, during venous occlusion for fourteen subjects.

	Measured Blood Flow (BF) (ml_{blood}/(100ml_{tissue})/min)					
**No.**	SD*I* (25 mm)	SD*I* (37 mm)	DS*I*	SDϕ (25 mm)	SDϕ (37 mm)	DSϕ
**1**	0.33 ± 0.006	0.24 ± 0.01	0.07 ± 0.01	0.24 ± 0.02	0.16 ± 0.03	0.02 ± 0.10
**2**	0.48 ± 0.01	0.36 ± 0.01	0.13 ± 0.03	0.37 ± 0.03	0.27 ± 0.05	0.1 ± 0.1
**3**	0.42 ± 0.01	0.35 ± 0.02	0.21 ± 0.03	0.44 ± 0.06	0.3 ± 0.1	0.2 ± 0.2
**4**	1.19 ± 0.03	1.09 ± 0.03	0.9 ± 0.1	1.17 ± 0.06	0.8 ± 0.1	0.3 ± 0.3
**5**	0.39 ± 0.03	0.37 ± 0.03	0.33 ± 0.05	0.59 ± 0.05	0.5 ± 0.1	0.3 ± 0.2
**6**	2.9 ± 0.2	2.6 ± 0.2	2.0 ± 0.1	1.9 ± 0.1	1.3 ± 0.1	0.28 ± 0.17
**7**	0.75 ± 0.03	0.72 ± 0.03	0.68 ± 0.04	0.68 ± 0.03	0.51 ± 0.03	0.2 ± 0.1
**8**	0.84 ± 0.03	0.82 ± 0.02	0.81 ± 0.04	0.80 ± 0.04	0.7 ± 0.1	0.5 ± 0.2
**9**	0.58 ± 0.04	0.71 ± 0.02	1.0 ± 0.1	0.74 ± 0.04	0.6 ± 0.1	0.3 ± 0.2
**10**	0.16 ± 0.02	0.21 ± 0.02	0.31 ± 0.05	0.37 ± 0.04	0.5 ± 0.1	0.6 ± 0.3
**11**	0.08 ± 0.01	0.11 ± 0.02	0.16 ± 0.04	0.22 ± 0.04	0.28 ± 0.1	0.4 ± 0.2
**12**	0.27 ± 0.01	0.34 ± 0.01	0.50 ± 0.02	0.47 ± 0.01	0.48 ± 0.02	0.50 ± 0.09
**13**	0.280 ± 0.003	0.346 ± 0.004	0.475 ± 0.008	0.36 ± 0.01	0.38 ± 0.02	0.39 ± 0.06
**14**	0.220 ± 0.02	0.260 ± 0.008	0.320 ± 0.007	0.40 ± 0.04	0.320 ± 0.007	0.20 ± 0.01

Table S3 Summary of the measured oxygen consumption (OC) obtained from single-distance intensity (SD*I*) at 25 and 37 mm, dual-slope intensity (DS*I*), single-distance phase (SDϕ) at 25 and 37 mm, and dual-slope phase (DSϕ), respectively, during arterial occlusion for twelve subjects. N/A indicates not applicable for subjects who did not undergo arterial occlusion or whose data were discarded due to large motion artifacts.

	Measured Oxygen Consumption (OC) (μmol_{O2}/(100ml_{tissue})/min)					
**No.**	SD*I* (25 mm)	SD*I* (37 mm)	DS*I*	SDϕ (25 mm)	SDϕ (37 mm)	DSϕ
**1**	2.48 ± 0.01	1.90 ± 0.01	0.7 ± 0.02	2.0 ± 0.1	1.6 ± 0.2	0.9 ± 0.2
**2**	1.30 ± 0.01	1.01 ± 0.01	0.55 ± 0.02	1.02 ± 0.01	0.82 ± 0.03	0.50 ± 0.06
**3**	5.23 ± 0.02	4.30 ± 0.02	2.45 ± 0.05	4.0 ± 0.1	2.1 ± 0.2	0.2 ± 0.2
**4**	1.49 ± 0.03	1.27 ± 0.02	0.81 ± 0.03	1.38 ± 0.02	0.97 ± 0.03	0.3 ± 0.1
**5**	2.169 ± 0.004	1.98 ± 0.01	1.61 ± 0.03	1.85 ± 0.02	1.4 ± 0.1	0.6 ± 0.2
**6**	N/A	N/A	N/A	N/A	N/A	N/A
**7**	N/A	N/A	N/A	N/A	N/A	N/A
**8**	1.45 ± 0.01	1.54 ± 0.01	1.72 ± 0.02	1.74 ± 0.02	1.44 ± 0.05	0.9 ± 0.2
**9**	3.78 ± 0.02	4.02 ± 0.02	4.50 ± 0.04	4.19 ± 0.03	3.1 ± 0.1	1.3 ± 0.2
**10**	3.2 ± 0.01	3.39 ± 0.02	3.90 ± 0.04	2.25 ± 0.04	1.6 ± 0.1	0.9 ± 0.5
**11**	2.35 ± 0.01	2.54 ± 0.01	2.63 ± 0.02	2.83 ± 0.03	2.4 ± 0.1	1.5 ± 0.2
**12**	4.30 ± 0.04	4.75 ± 0.05	4.87 ± 0.06	4.2 ± 0.1	3.3 ± 0.1	1.8 ± 0.3
**13**	0.73 ± 0.01	0.82 ± 0.01	0.98 ± 0.02	0.64 ± 0.01	0.55 ± 0.02	0.4 ± 0.1
**14**	0.60 ± 0.01	0.65 ± 0.01	0.74 ± 0.01	0.70 ± 0.01	0.60 ± 0.01	0.5 ± 0.1

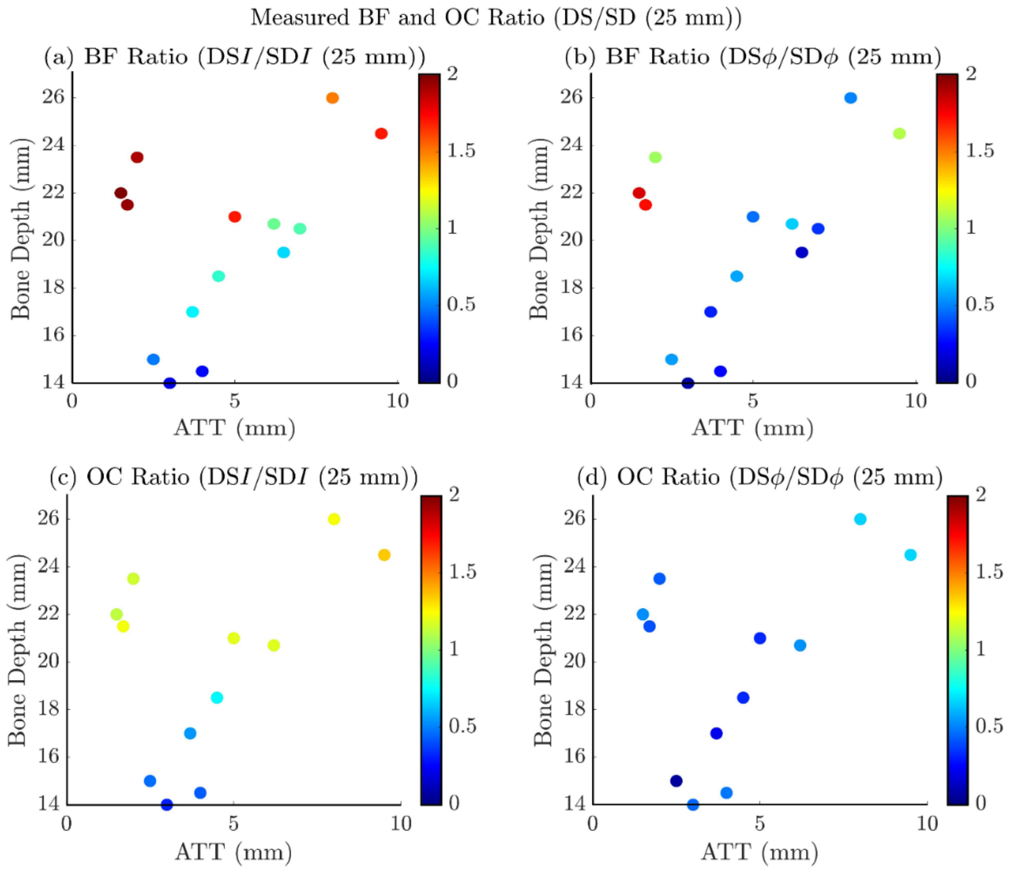

Fig. S1 Ratios of measured blood flow (BF) and oxygen consumption (OC) across all subjects, obtained from dual-slope (DS) relative to those at shorter single-distance (SD) at 25 mm, shown as DS/SD (25 mm), and plotted as a function of bone depth and adipose tissue thickness (ATT). (a) BF ratio for intensity (I) data; (b) BF ratio for phase (ϕ) data; (c) OC ratio for intensity (I) data; (d) OC ratio for phase (ϕ) data.

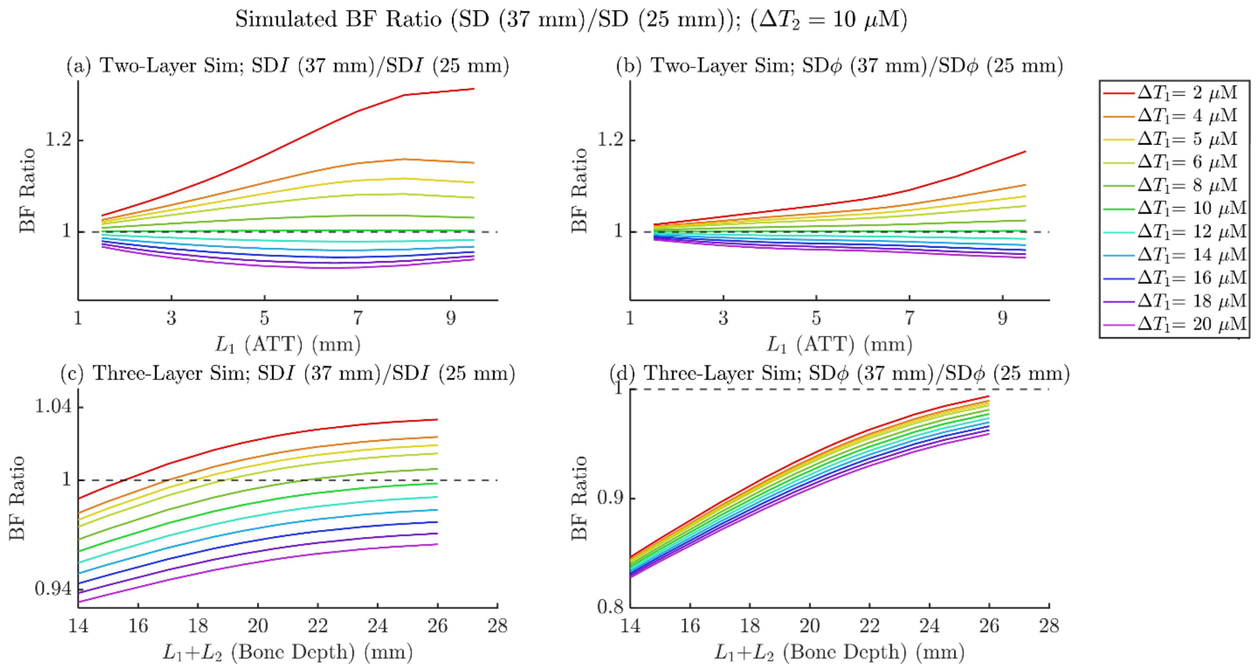

Fig. S2 Theoretical simulations of the ratios of blood flow (BF) obtained by SDI data at 37 mm over BF obtained by SDI data at 25 mm (BF_{SDI} (37 mm)/BF_{SDI} (25 mm)) and the ratios of BF obtained by SDϕ data at 37 mm over BF obtained by SDϕ data at 25 mm ($BF_{SD\phi}$ (37 mm)/$BF_{SD\phi}$ (25 mm)) for the two-layer model as a function of L_1 (or adipose tissue thickness (ATT)) (panels (a) and (b)) and for the three-layer model as a function of $L_1 + L_2$ (or bone depth) (panels (c) and (d)). ΔT_1 was varied within the range of 2-20 μM, ΔT_2 was set to 10 μM, and the baseline optical properties were those reported in Table 2. L_1 was set to 1.5 mm for the three-layer model.

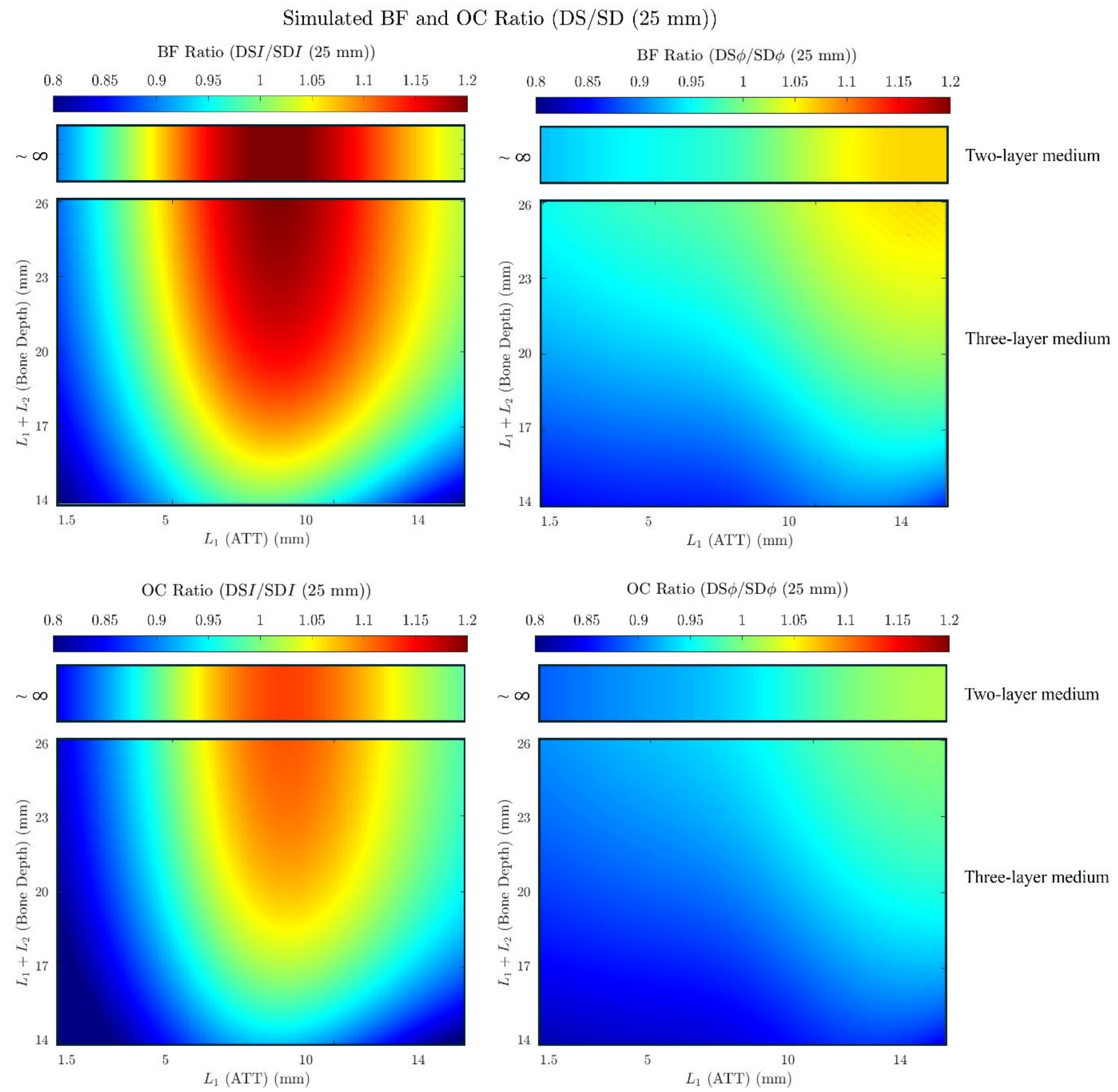

Fig. S3 Ratios of simulated blood flow (BF) and oxygen consumption (OC) obtained from dual-slope (DS) relative to those at shorter single-distance (SD) at 25 mm, shown as DS/SD (25 mm), and plotted as functions of adipose tissue thickness (L_1) and bone depth ($L_1 + L_2$). (a) BF ratio for intensity (I) data; (b) BF ratio for phase (ϕ) data; (c) OC ratio for intensity (I) data; (d) OC ratio for phase (ϕ) data. Each subfigure shows results for both a two-layer model (for which $L_1 + L_2 \sim \infty$) and a three-layer model. For blood flow simulations, ΔT_1, ΔT_2, and ΔT_3 were set to 5 μM, 10 μM, and 0 μM, respectively. For oxygen consumption simulations, ΔD_1, ΔD_3, and ΔD_3 were set to 5 μM, 10 μM, and 0 μM, respectively. For all simulations, μ_a and μ_s' for each layer (Layer 1, Layer 2, and Layer 3, respectively) were set to μ_a = (0.0083, 0.0166, 0.0083) mm^{-1} at λ = 690 nm, μ_a = (0.0104, 0.0208, 0.0104) mm^{-1} at λ = 830 nm, μ_s' = (1, 0.5, 1.5) mm^{-1} at λ = 690 nm, and μ_s' = (0.98, 0.42, 1.3) mm^{-1} at λ = 830 nm (see Table 2).
